# Disentangling How Climate and Dispersal Drive Temporal Trends in Synchronous Population Dynamics

**DOI:** 10.1002/ece3.71443

**Published:** 2025-05-26

**Authors:** Lisbeth A. Hordley, Gary D. Powney, Tom Brereton, Simon Gillings, Owen L. Petchey, David B. Roy, Joseph A. Tobias, James Williams, Tom H. Oliver

**Affiliations:** ^1^ School of Biological Sciences University of Reading Reading Berkshire UK; ^2^ Butterfly Conservation Wareham Dorset UK; ^3^ UK Centre for Ecology & Hydrology Wallingford Oxfordshire UK; ^4^ British Trust for Ornithology Thetford UK; ^5^ Department of Evolutionary Biology and Environmental Studies University of Zurich Zurich Switzerland; ^6^ Department of Life Sciences Imperial College London Ascot Berkshire UK; ^7^ Joint Nature Conservation Committee Peterborough UK

**Keywords:** dispersal, long‐term monitoring data, Moran effect, population synchrony

## Abstract

Spatially synchronised population dynamics are driven by a combination of shared environmental conditions among sites and the movements of individuals between sites. Untangling the drivers of population synchrony requires investigation of how populations are correlated across space and time in relation to climate and mobility‐related attributes. Here, we use species survey data from over four decades to investigate average levels and temporal trends in population synchrony for 58 British bird and butterfly species. We first show that population synchrony is significantly associated with synchrony in seasonal climatic variables. After accounting for spatiotemporal climatic patterns, we determine whether temporal trends in population synchrony are shaped by mobility‐related attributes. We test this through an interspecies comparison using three variables correlated with mobility: biotope specialism, estimated species mobility, and local abundance change, which is known to affect emigration rate. We find that temporal trends in population synchrony are most marked for generalist butterfly species, butterflies with high estimated mobility, and butterflies that had changed in their mean abundance. For birds, we find changes in population synchrony are associated with specialist bird species and those that increased in abundance over time. Our results reveal a widespread effect of mobility attributes and abundance patterns on population synchrony over time, suggesting that variation in dispersal is a key factor determining the extent to which population dynamics are synchronised.

## Graphical abstract

Spatially synchronised population dynamics are driven by a combination of shared environmental conditions among sites and the movements of individuals between sites. We were able to disentangle these drivers and show that after trends in climate autocorrelation were accounted for, population synchrony is significantly associated with mobility‐related attributes of birds and butterflies.

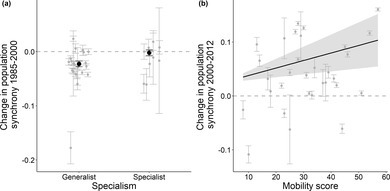

## 1 Introduction

Population synchrony, measured as the correlation in abundance between spatially separated populations over time, is exhibited in many taxonomic groups including insects (Sutcliffe et al. 1996), fish (Cheal et al. 2007), birds (Bellamy et al. 2003; Kerlin et al. 2007; Paradis et al. 2000), plants (Kiviniemi and Löfgren 2009), and mammals (Swanson and Johnson 1999). Synchronous population dynamics can be driven by a variety of factors, including dispersal (Ripa 2000), environmental factors (Moran 1953; Ranta et al. 1997), and trophic interactions (Ims and Andreassen 2000). Spatial synchrony is thought to be important for the long‐term viability of metapopulations as synchronised population dynamics can prevent poorly performing populations from being rescued and can increase the risk of meta‐population extinction (Heino et al. 1997). Therefore, it is crucial to measure how spatial population synchrony is changing across time, as well as whether such changes can be attributed to the two key drivers, dispersal and climate.

Previous research has shown theoretical and empirical support that shared environmental conditions drive population synchrony, that is, the ‘Moran effect’ (Grenfell et al. 1998; Moran 1953). The effect declines with increasing distance between populations partly due to spatial autocorrelation in climatic conditions (Hanski and Woiwod 1993; Powney et al. 2011; Roland and Matter 2007). Additional research has shown that populations are more synchronised if they occupy similar habitat types (Powney et al. 2010, 2011) or are situated at geographic range margins (Mills et al. 2017; Powney et al. 2010), which can lead to increased climatic constraints on marginal populations, reducing the availability of suitable microhabitats (Oliver et al. 2014; Powney et al. 2010). Several studies have concluded that climate is a major driver of temporal trends in population synchrony (Black et al. 2018; Hansen et al. 2020; Sheppard et al. 2016; Shestakova et al. 2016). For example, climate change could be driving an increased frequency of extreme weather events, leading to greater synchronised population dynamics (Black et al. 2018). In addition, there may be temporal trends in the degree of spatial autocorrelation in climate (Post and Forchhammer 2004).

Movement of individuals between populations also leads to increased synchrony in population dynamics (Hanski 1998; Ranta et al. 2008; Wanner et al. 2024). Density‐dependent emigration of individuals can link populations, leading to fluctuations in population synchrony (Ranta et al. 1995). Empirical evidence shows that population dynamics in different locations are more synchronised for species with high estimated dispersal ability (as measured using mark‐release‐recapture (Bellamy et al. 2003; Paradis et al. 1999); expert opinion (Sutcliffe et al. 1996); or using dispersal‐related traits as a proxy for dispersal ability (Tittler et al. 2009)). There is also evidence showing that average abundance of species, measured at both the local and regional scale, is also associated with the degree of population synchrony (Bellamy et al. 2003; Paradis et al. 1999, 2000), suggesting that more abundant species have higher ‘propagule pressure’ (emigration of individuals) facilitating the spread of populations (Hanski 1998). Moreover, population synchrony is related to landscape suitability (Powney et al. 2011, 2012), demonstrating the sensitivity of population synchrony to the movement of individuals. Other evidence has shown that residual synchrony (after accounting for range position and habitat context and various other factors) reflects actual movements of individuals measured using mark‐release‐recapture (Oliver et al. 2017).

Several studies have attempted to disentangle the role of dispersal on population synchrony from that of shared environmental conditions (known as the ‘Moran effect’) (Lande et al. 1999; Liebhold et al. 2004; Saether et al. 2007; Haynes et al. 2013). Kendall et al. (2000) found strong interactions between dispersal and the correlated environment, which cause greater synchrony between populations. Therefore, understanding trends in population in synchrony over time, and how this relates to species' dispersal traits, needs to consider the effects of climatic autocorrelation. To our knowledge, there has not yet been an attempt to disentangle dispersal and climate with regard to *temporal* trends in population synchrony after accounting for the direct impact of the Moran effect.

To investigate this, we calculate both average levels and temporal trends in population synchrony for 58 British birds and butterflies using long‐term monitoring datasets from 1980 to 2016 for a total of 3306 sites across Great Britain. We use data from three monitoring schemes: the UK Butterfly Monitoring Scheme (UKBMS), the Common Birds Census (CBC), and the Breeding Bird Survey (BBS). We develop approaches to account for spatiotemporal climatic patterns that drive correlated population dynamics, that is, a ‘dynamic’ Moran effect, whereby temporal trends in spatial autocorrelation of climate are driving temporal population synchrony. After accounting for these effects of climate, we produce a residual temporal trend in population synchrony that we hypothesise is related to three movement‐related species attributes: specialism, mobility, and population abundance. This is then tested through an interspecies comparison where we predict that certain types of species differ in average and temporal levels of population synchrony.

Our selected species attributes all relate to the movement of species across the landscape. Ecological theory suggests that specialists are less mobile compared to generalists, which may be due to their resource patches being more sparsely distributed, therefore leading to selection for lower dispersal (Jocque et al. 2010; Stevens et al. 2014) or to avoid competition with generalists that have higher dispersal rates and are able to access resources unavailable to specialists (Nagelkerke and Menken 2013). Empirical evidence supports these theories, showing that specialists have lower dispersal capacity than generalists (Verberk et al. 2010; Funk et al. 2013; Dapporto and Dennis 2013; Kneitel 2018). An additional explanation demonstrates that generalists are better able to move across a poor quality, fragmented landscape compared to specialists (Ramiadantsoa et al. 2018). This has been shown in butterflies, whereby species with traits associated with generalists including higher dispersal ability were more common in intensified grasslands (Börschig et al. 2013). The size of populations can also relate to the movement of species through positive density‐dependent emigration. Evidence for positive density‐dependent dispersal exists for birds (Matthysen 2005) and butterflies (Enfjäll and Leimar 2005; Nowicki and Vrabec 2011) whereby emigration at high densities allows offspring fitness to increase or to reduce competitive interactions.

Based on the evidence presented here, we predict that generalist species, with higher mobility and higher mean abundance, will have higher average levels of population synchrony due to greater movement frequency between locations (Table 1). We also predict that species increasing abundance over time will show increases in population synchrony over time due to increased emigration of individuals. However, we make no a priori hypotheses on the relationship between mobility and specialism and change in synchrony over time due to a lack of previous evidence, as well as the fact that these relationships are likely to be highly contingent on the level of fragmentation in the landscape.

**TABLE 1:** Table of hypotheses and model results for species attributes in relation to both average levels and temporal trends in synchrony. Ticks indicate whether a hypothesis was supported by our analysis, dashes represent unsupported hypotheses (i.e., non‐significant in the direction predicted), and crosses represent when the hypothesis was unsupported and the opposite result was found by our analysis. Symbols in parentheses indicate a supported hypothesis for one time period only. Grey boxes represent instances where no a priori hypotheses were made.

Explanatory variable	Response variable	Hypothesis	Met with hypothesis?			Direction of effect found		
			UKBMS	CBC	BBS	UKBMS	CBC	BBS
Biotype specialism	Average synchrony	Generalists have higher levels of average synchrony	**—**	**—**	**—**	No significant relationship	No significant relationship	No significant relationship
	Change in synchrony					Generalists decline in synchrony more steeply between 1985 and 2000 and increase in synchrony more steeply between 2000 and 2012 compared to specialists	Specialists show a more positive change in synchrony over time compared to generalists	Specialists show a more positive change in synchrony over time compared to generalists
Mobility	Average synchrony	More mobile species have higher average levels of synchrony (Bellamy et al. 2003; Paradis et al. 1999; Sutcliffe et al. 1996)	(✓)	**—**	**—**	More mobile species have higher average levels of synchrony	No significant relationship	No significant relationship
	Change in synchrony					More mobile species show greater increase in synchrony over time between 2000 and 2012	No significant relationship	No significant relationship
Average abundance	Average synchrony	More common species have higher average levels of synchrony (Bellamy et al. 2003; Paradis et al. 1999, 2000)	**—**	✓	**—**	No significant relationship	More common species have higher average levels of synchrony	No significant relationship
Species' abundance change over time (categorical variable including non‐significant trends)	Change in synchrony	Species increasing in abundance also increase in synchrony over time	(✓)	**—**	**—**	Butterflies increasing in abundance increased in synchrony more rapidly between 2000 and 2012 compared to those declining in abundance	No significant relationship	No significant relationship
Species' abundance change over time (categorical variable, significant trends only)	Change in synchrony	Species increasing in abundance also increase in synchrony over time	✓✕	✓	**—**	Species increasing in abundance increase in synchrony more rapidly between 1985 and 2000, but species declining in abundance increase in synchrony more rapidly between 2000 and 2012	Species increasing in abundance show greater increase in synchrony over time	No significant relationship

## 2 Materials and Methods

### 2.1 Data Collation

Butterfly data were derived from the UKBMS (Pollard and Yates 1993). UKBMS transects are walked by trained volunteers who survey 5 m‐wide strip transects for each of 26 weeks between April and September, recording all butterflies observed. Further details can be found in Pollard and Yates (1993) and Rothery and Roy (2001). An index of abundance for each butterfly species for each transect, each year from 1980 to 2016, was extracted from the UKBMS database. To ensure adequate data for analysis, resident butterfly species which had at least 75% of years with 50 or more sites sampled per year were included in the analysis.

Woodland bird abundance data were derived from two datasets, the CBC and the BBS. The CBC monitoring scheme monitored population trends for British breeding birds from 1962 until 2000 (Marchant et al. 1990). Volunteer observers undertook repeated surveys between 8 and 10 times a year between late March and early July, recording all species seen or heard at each site. The BBS has monitored birds since 1994, where two 1 km transects are visited twice a year, once between April and mid‐May (early visit), and once between mid‐May and the end of June (late visit) and all birds seen or heard are recorded (Harris et al. 2018). The total number of adult birds of each species for each site and each visit is calculated for each year. We obtained the maximum number of adult birds across all visits at each site for the years 1980–2000 from the CBC and 1994–2016 from the BBS. Species which had at least 75% of years with 50 or more sites sampled per year were included in the analysis.

In addition to interannual fluctuations in population size, raw abundance values also reflect long‐term temporal trends arising from drivers such as land use and climate change; therefore, we used rates of change to focus on interannual population synchrony (Bjørnstad et al. 1999). We converted annual abundance values into rates of change as follows: *logN*_*t*_ − *logN*_*t‐1*_, where *N*_*t*_ is the abundance index estimate at time *t* (Powney et al. 2010). We added one to all population counts prior to the growth rate calculation to avoid taking the log of zero.

### 2.2 Population Synchrony

For each species, population synchrony between pairs of monitoring sites was estimated using the Pearson's correlation coefficient of yearly population growth rates. To assess temporal trends in population synchrony, we repeatedly calculated population synchrony using a 10‐year moving window (Bjørnstad et al. 1999). A 10‐year moving window was selected to balance the need for a reasonable‐length time series to estimate population synchrony versus the number of separate windows where we could calculate population synchrony. The following pair‐wise site combinations were excluded from the analysis: (i) for any pair of sites, less than 7 years of growth rates in common, to ensure data quality; (ii) if either site had a zero abundance count followed by a positive abundance count, to avoid population synchrony being calculated where there are a chain of zeros followed by positive values (associated with new site colonisation) as this can inflate synchrony values and increase Type I errors (Sutcliffe et al. 1996); and (iii) site combinations that were more than 100 km apart. Although evidence has shown synchrony remains positively associated with landscape suitability for sites up to 200 km apart (Powney et al. 2011), we selected an upper distance limit of 100 km for computational feasibility. Additionally, due to computational limitations, synchrony was only calculated on BBS sites with at least 10 years of non‐consecutive data, and for a maximum of 10,000 random pairs of sites. This represents a subset of pairs of sites, with the extent proportion of all site pairs ranging among species from 3% to 98% of total available site comparisons (with a median of 14%). We repeated this process five times to confirm that each subset adequately represented the whole dataset, and results were very consistent between sub‐samples (Figure S2). The resulting dataset had population synchrony values for 32 butterflies from 701 sites between 1980 and 2016, 26 birds from 106 sites between 1980 and 2000, and 24 birds from 2499 sites between 1994 and 2016 (Tables S1–S3).

### 2.3 Climate Synchrony

To determine whether temporal trends in population synchrony are driven by patterns in climatic synchrony over time, we measured synchrony of mean temperature and mean precipitation for each season (i.e., eight variables) using 5 km gridded climate data from Met Office et al. (2017). We converted population synchrony sites from 1‐ to 5‐km grid squares and matched these to climate data for each of the three datasets. Synchrony was calculated using the same method as population synchrony (aside from calculating growth rates), that is, calculating a Pearson's correlation metric for each climate variable between each pair of monitoring sites for grid squares using a 10‐year moving window. The resulting dataset had climate synchrony values from 686 UKBMS sites from 1980 to 2016, 106 CBC sites from 1980 to 2000, and 2490 BBS sites from 1994 to 2016 (Figure S1).

### 2.4 Control Variables

To control for climate‐related variables that can influence population synchrony, three attributes were calculated for each pair of sites, in each dataset, to include as covariates in our statistical models. First, sites that are closer together have higher levels of synchrony (Hanski and Woiwod 1993); therefore, distance was calculated as the Euclidean distance (km) between each pair of sites. Second, populations at the northern (cold) range margin are more synchronised (Powney et al. 2010). As a proxy for range position, we estimated mean northerliness, which was calculated as the mean Northing (km from Ordnance Survey National Grid) between each pair of sites. Finally, sites with similar habitat types are more synchronised due to similarities in local microclimate conditions (Powney et al. 2010). To estimate habitat similarity between sites, we used a Renkonen's percentage similarity index of a 500 m buffer surrounding each of the sites in a pair (Jost et al. 2011; Renkonen 1938). The index was bound between 0 and 1, with a value of 1 for two sites surrounded by the same habitat composition, and 0 being completely distinct compositions. Habitat data were extracted from the CEH Land Cover Map 2007 (Morton et al. 2011) and aggregated to the broad habitat level (10 habitat biotopes in total). Sites for CBC were primarily woodland sites, with woodland type recorded as a categorical variable (four types); therefore, habitat similarity was calculated as a binary variable, with 1 representing a pair of sites with the same woodland type and 0 representing a pair of sites with different woodland types.

### 2.5 Species Attributes

We selected three species attributes: biotype specialisation, mobility, and abundance, to relate to levels of population synchrony. For biotype specialisation, butterflies were split into either wider countryside or habitat specialist species (Asher et al. 2001) and birds were classified into either woodland generalists or specialists (Defra 2017). Mobility ranks for butterflies were obtained from Wilson et al. (2004) and breeding dispersal distances for birds were taken from Paradis et al. (1998). We obtained two measures of abundance: average abundance and change in abundance over time. Our measure of average abundance for butterflies uses the Wider Countryside Butterfly Survey (Brereton et al. 2011), which has run since 2009, where volunteers visit sites two to four times a year counting butterflies along two parallel 1 km transects. Although they show similar trends, the WCBMS abundance estimates are deemed more representative of the whole landscape compared to the UKBMS sites (Brereton et al. 2011). We therefore used WCBMS to estimate average abundance of each butterfly species across our study region (Great Britain) by calculating the mean abundance for each species between 2009 and 2016. For birds, we used national population estimates from Musgrove et al. (2013). The change in abundance for butterflies uses the UKBMS Collated Index data, which is a national annual index for each species for each year derived using a statistical model (Moss and Pollard 1993; Rothery and Roy 2001). We calculated the mean difference in abundance between two independent 10‐year windows: 1980–1989 and 1995–2004 to represent the change in abundance for the first two decades, and between 1995–2004 and 2007–2016 for the latter two decades for each species. For the CBC, we calculated the mean difference in abundance between 1980–1989 and 1991–2000 for each species. For the BBS, we calculated the mean difference in abundance between 1994–2003 and 2007–2016 for each species. All species were first classified as either increasing or decreasing in abundance over time regardless of significance. We also used a *t* test to determine whether species had significantly increased or decreased in abundance and removed species, which showed no significant change in abundance over time. The analysis was run on both non‐significant abundance changes and significant abundance changes. Mean abundance change was treated as a categorical variable in this analysis because absolute change values are not easily comparable between species without additional information on starting values, and they are also susceptible to bias from differences in detectability between species (Isaac et al. 2011; Johnston et al. 2014). Attribute data were missing for 12 species; see Tables S1–S3 for a list of all species and associated attributes.

### 2.6 Statistical Analysis

#### 2.6.1 Accounting for Climatic Synchrony

Initially, we sought to account for variation in population synchrony that could be attributed to climate synchrony. We found no evidence for collinearity between each climate synchrony variable for each dataset; therefore, we fitted mixed effects models using the *lme4* package (Bates et al. 2015) to each of the three monitoring datasets separately (‘all species models’). Each model contained population synchrony values for every pair‐wise site comparison for each species as the response variable, and distance, habitat similarity, mean northerliness and the mid‐year of each moving window as continuous fixed effects to account for the influence of these on synchrony, along with each of the eight climate synchrony variables as continuous fixed effects. Species and pair ID of the sites were included as random intercepts to account for repeated measures and the number and identity of monitoring sites varying through time. Any climate variable with a significant relationship with population synchrony (*p* < 0.05) was included as a covariate in future analyses to account for climatic effects. We note that this approach could be conservative as we may be less likely to detect other patterns in population synchrony than if we had attempted to avoid any possible overfitting. Since synchrony measures of pair‐wise sites are not independent, to obtain *p*‐values we ran 1000 permutation tests (e.g., see Powney et al. 2012) to determine the significance of change in climate synchrony over time. At each permutation, the predictor variable (climate variable) was randomised using the sample function in R, and a linear mixed effects model fitted, and the *F*‐values extracted. We plotted the frequency distribution of the *F*‐values and calculated the *p*‐values for each predictor variable based on the position of the observed versus simulated values (e.g., a value in the top 5% of the *F*‐value frequency distribution would have a significant *p*‐value of < 0.05).

#### 2.6.2 Population Synchrony and Species Attributes

To understand whether species attributes could explain differences in population synchrony between species, we fitted a variant of the all‐species models for each dataset by including distance, habitat similarity, mean northerliness, and the significant climate synchrony variable(s) (unique for each dataset) as continuous fixed effects to account for known drivers of population synchrony. Each species attribute was included as an additional fixed effect, and as attribute data were missing for some species (Tables S1–S3), each attribute was placed into three separate models, one model for each attribute (biotope specialism as a fixed categorical effect and mobility and average abundance as fixed continuous effects). All continuous fixed effects were standardised to zero mean and one standard deviation. To ensure that population synchrony was not being driven by phylogenetic relatedness, we tested for an additional effect of family and genus in the all‐species models for each dataset. We did not find a significant result for butterflies or BBS birds, but family was significant for CBC birds. Hence, this variable was added as a random effect unless we obtained singular fit errors, where we removed the family random effect and found no qualitative difference in results between the two models. We also included an interaction between the mid‐year of the moving window (as a continuous variable) and each species attribute in separate models for each attribute. This determined whether certain types of species were increasing or decreasing in population synchrony between two non‐overlapping 10‐year periods. This was repeated using abundance change categories obtained from both the non‐significant abundance changes and significant abundance changes over time. As above, we conducted 1000 permutation tests (e.g., see Powney et al. 2012) on each species to determine the significance of change in synchrony between the two comparison years. At each permutation, the predictor variable (species attribute) was randomised using the sample function in R, a linear mixed effects model fitted, and the *F*‐values extracted. We plotted the frequency distribution of the *F*‐values and calculated the *p*‐values for each predictor variable based on the position of the observed versus simulated values (e.g., a value in the top 5% of the *F*‐value frequency distribution would have a significant *p*‐value of < 0.05).

All models are described using mathematical notation in Appendix S2.

All statistical analysis was carried out using R 4.1.0 (R Core Team 2023).

## 3 Results

### 3.1 Climate Accounts for Variation in Population Synchrony

We selected which climate synchrony variables explained a significant amount of variation in population synchrony for each dataset. For UKBMS, we selected seven climate synchrony variables, as only summer temperature was non‐significant (*R* ^2^ = 0.0079; Table S4). For CBC birds, only summer temperature was included (*R* ^2^ = 0.00014) and for BBS birds, autumn temperature, and spring and winter rainfall were selected (*R* ^2^ = 0.000024; Table S4). These variables remained significant after permutation tests were run (Table S5) and were included in all future models as fixed effects to account for the relationship between climate and population synchrony.

### 3.2 Associations With Species Attributes

**FIGURE 1:**
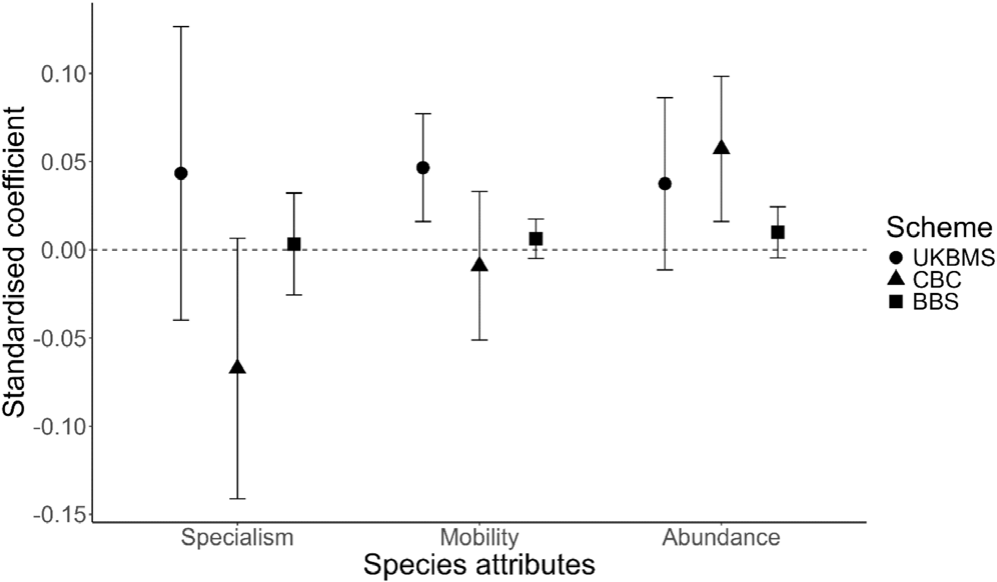
Standardised regression coefficients from mixed effects models with average synchrony as the response variable and species attributes as fixed effects. Symbols mark the regression coefficients for each fixed effect and error bars mark the 95% confidence intervals. A positive coefficient indicates that a higher level of a given species attribute (e.g., higher mean species abundance) is associated with greater synchrony in population dynamics between sites for that species.

**FIGURE 2:**
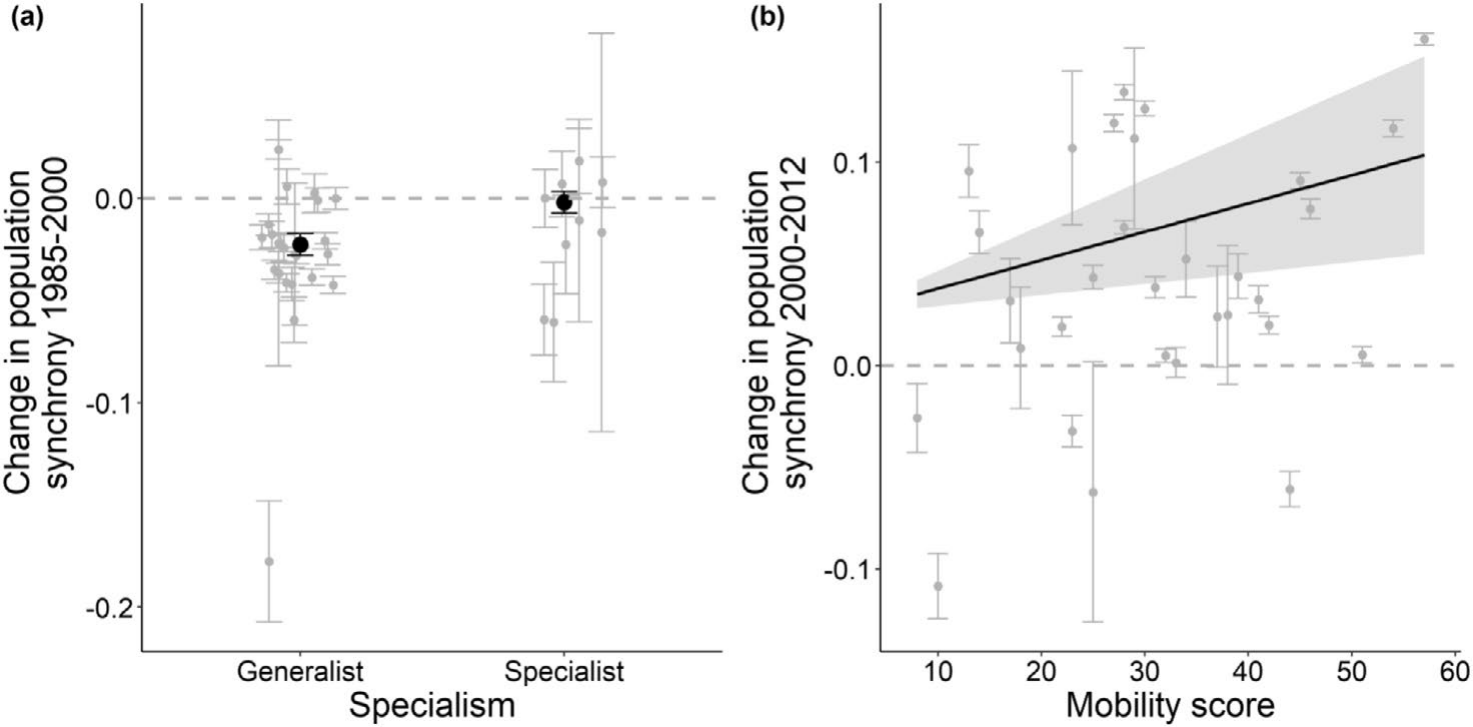
The change in population synchrony over time for butterflies in relation to (a) biotype specialism and (b) mobility. Dashed grey lines represent zero change in population synchrony over time, grey points represent each species raw data with standard error bars, and black points represent the slope (i.e., change in synchrony over time) from the mixed effects models with their associated standard errors. The solid line represents the slope (i.e., change in synchrony over time) for each mobility score from the mixed effects models with the associated standard error. Grey points were scattered horizontally randomly with a small deviation to increase clarity.

With regard to biotope specialism, we found no significant relationship with average levels of synchrony for butterflies or birds (Figure 1; Table 1). For butterflies, we found a significant association of specialism and temporal trend in synchrony, with generalists showing a greater decline in synchrony between 1985 and 2000 compared to specialists (Figure 2a) and showing the greatest recovery in synchrony between 2000 and 2012 (Figures S3 and S4; Table S6). For BBS birds, we found that specialist birds showed an increase in synchrony compared to generalists under the BBS dataset (Figures S3 and S5; Table S8). Similarly, CBC specialist species increased in synchrony between 1985 and 1996, whereas generalists showed a small decline (Figures S3 and S6; Table S7).

With regard to estimated mobility of species, our analysis also showed that more mobile butterfly species had higher average levels of synchrony, while average synchrony for birds was not related to dispersal ability (Figure 1). For BBS birds, we found that species with a high dispersal distance show marked increases in synchrony over time (Figure S7; Table S8). However, this result was primarily driven by four species with high dispersal distances (blackcap, robin, willow warbler, and wren), and after these were removed, the relationship was non‐significant (Table S8). For butterflies, we found that the recovery in synchrony between 2000 and 2012 was greatest for butterflies with high mobility (Figure 2b; Table S6).

**FIGURE 3:**
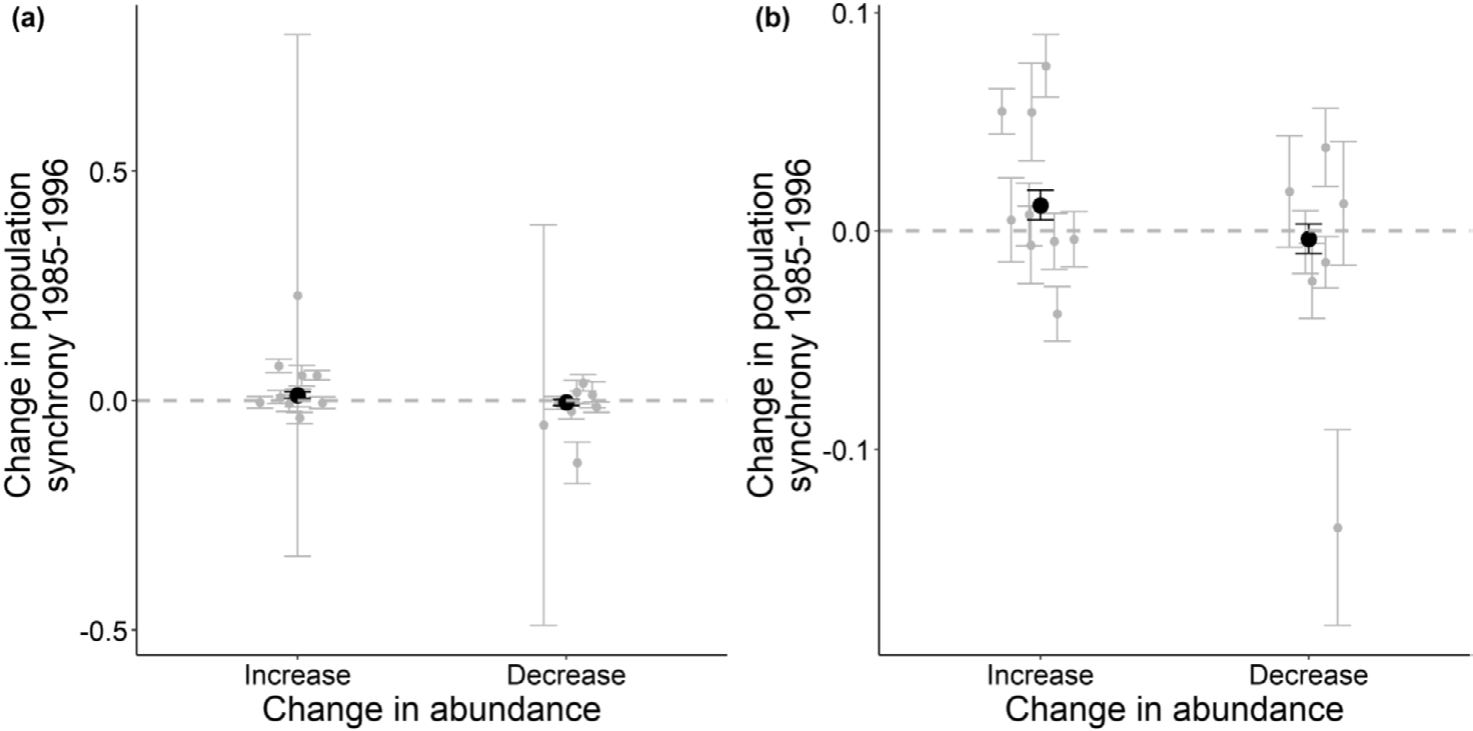
Change in population synchrony over time for CBC birds in relation to significant changes in abundance between 1985 and 1996 with (a) all species with significant changes in abundance (*n* = 19) and (b) two species with high variance around the raw data removed (*n* = 17). Dashed grey lines represent zero change in population synchrony over time, grey points represent each species raw data with standard error bars, and black points represent the slope (i.e., change in synchrony over time) from the mixed effects models with their associated standard errors. Grey points were scattered horizontally randomly with a small deviation to increase clarity.

In relation to mean abundance of species, we found that more common CBC birds had higher average levels of population synchrony, but we did not find any significant effects for BBS birds or butterflies (Figure 1). Analysing species that showed non‐significant changes in abundance over time, we found that butterflies which increased in abundance also increased in population synchrony between 2000 and 2012 more rapidly than those which declined in abundance (Figure S8). We did not find any significant results for birds (Tables S7 and S8). When only using species with significant changes in abundance over time, we found that butterflies that had significantly declined in abundance over time had decreased in synchrony over time between 1985 and 2000, whereas species that had significantly increased in abundance showed little change (Figure S9). Between 2000 and 2012, all species increased in synchrony, but those that showed significant declines in abundance increased in synchrony more rapidly than those declining in abundance (Figure S10). For CBC birds, species with significant increases in abundance over time also increased in population synchrony faster than those showing significant decreases in abundance (Figure 3a); however, this result was non‐significant when two species (redstart and lesser whitethroat) with high variance were removed (Figure 3b; Table S7). We found no significant result for BBS using significant changes in abundance over time (Table S8). All species attribute results remained significant after permutation tests were run (Table S9). A summary of these results in relation to whether they support a priori hypothesis can be found in Table 1.

## 4 Discussion

These analyses provide new evidence for the relative role of dispersal in population synchrony trends while accounting for climatic effects. Our results show that trends in population synchrony are clearly driven to some extent by climatic factors, including both temperature and rainfall. This is interesting as several studies have suggested that the spatial scaling of environmental variables known to influence local population dynamics is often far larger than the scaling of the synchrony in population dynamics (Koenig 2002), and that dispersal or density regulation are likely more important in creating such synchronous fluctuations than any spatial covariation that exists in the environment (Lande et al. 1999; Saether et al. 2007). In contrast, other studies have shown parallel increases in population synchrony and environmental synchrony, suggesting a potential role of the Moran effect in driving shared population dynamics over time (Kahilainen et al. 2018; Koenig and Liebhold 2016; Sheppard et al. 2016; Shestakova et al. 2016).

Our analysis supports the view that trends in climatic autocorrelation can be important in driving population synchrony. For butterflies, all bar one of the eight climate variables tested were individually associated with population synchrony, whereas for the BBS dataset, only three variables were significant and for CBC birds only summer temperature (Table S4). Such trends in climate autocorrelation that we find here, suggest that there is presence of a ‘dynamic’ Moran effect, whereby temporal trends in climate synchrony drive temporal trends in population synchrony, as opposed to spatial trends alone. Additional research has shown that monitoring sites that share similar habitat, and are situated closer to a geographic range margins have higher mean synchrony values (Hordley et al. 2022; Powney et al. 2010; Roland and Matter 2007; Sutcliffe et al. 1996), providing further evidence of the role that shared climate plays in driving population synchrony.

Once trends in climatic autocorrelation (the ‘dynamic’ Moran effect) were accounted for, we found several clear relationships between population synchrony and mobility‐related attributes. Generalist butterflies showed the greatest changes in population synchrony over time, demonstrating the greatest decline in synchrony in the first two decades of our data (Figure 2a), followed by the most rapid recovery in the latter two decades. Generalist butterflies have increased their geographic distribution in recent years (Warren et al. 2001) and may be responsive to changes in the wider landscape because their host plants occur more widely. For example, landscape context up to 10 km around sites has been shown to be important in influencing the population dynamics of generalist butterflies, while specialist species respond to more localised aspects of landscape structure (Oliver et al. 2010). As such, generalist butterflies tend to be more mobile, and so changes in population synchrony for generalist butterflies may be due to their greater ability to move across landscapes. In contrast, we found that specialist birds showed a different pattern, with increases in synchrony across our study period (Figures S5 and S6). Most specialist bird species in our study are also migrants, which spend winter months in Europe and Africa (e.g., blackcap and chiffchaff) (Hewson and Noble 2009). Migrant species have been shown to disperse further than resident species (Martin and Fahrig 2018; Paradis et al. 1998); population synchrony was found to be higher in short‐distance migrants compared with resident species (Martin et al. 2023). Therefore, migrants could show greater increases in population synchrony over time as population synchrony is being driven by movement through migration, as opposed to local movements. Alternatively, studies have shown that climate autocorrelation on non‐breeding grounds at particular seasons, influences the population synchrony of migratory species (Martin et al. 2023; Walter et al. 2020); however, we did not assess the trends in climate autocorrelation in non‐breeding grounds here.

Regarding the mobility of species, we find that more mobile butterflies have higher average levels of population synchrony, providing further evidence that dispersal is a key driver of shared population dynamics (Bellamy et al. 2003; Chevalier et al. 2014; Paradis et al. 1999, 2000; Sutcliffe et al. 1996). After an overall decline in the synchrony of most butterflies between 1985 and 2000, the recovery of synchrony was most marked in more mobile butterflies across the latter period of our study (Figure 2b), a similar result to those found for generalist butterflies (Table S6). This could be a context‐dependent response to environmental factors, where the landscape structure and/or climate conditions benefit more mobile generalist species but hinder less mobile species. It also fits with the recent historical pattern of landscape‐level restoration of common habitats benefitting mobile generalist species through the wide uptake of agri‐environment schemes, yet a concurrent decline in rarer habitats such as lowland heathland and calcareous grassland meadows that are used by less mobile and more specialised species (Carey et al. 2008). We also found that birds with higher dispersal distance have increased in synchrony across the latter two decades, whereas species with a lower dispersal distance have declined in synchrony (Figure S7). However, this result was primarily driven by four migrant species: blackcap, robin, willow warbler, and wren. After removing these species, we found no relationship between dispersal distances and the temporal trend in synchrony (Table S8).

Regarding the abundance of species, we found that more common birds show higher levels of average synchrony, as has been shown previously (Bellamy et al. 2003; Paradis et al. 1999, 2000). However, despite finding that more abundant species (i.e., with higher mean abundance across the study region) are more synchronous (Figure 1), we cannot determine whether this is due to greater movement rates or due to smaller populations being more sensitive to demographic stochasticity and therefore less synchronous, or due to a lower influence of sampling variance on larger populations (Ims and Andreassen 1999; Freckleton et al. 2006; Santin‐Janin et al. 2014). Additionally, we found that butterflies that show increases in abundance over time (including non‐significant trends) have shown strong increases in population synchrony during the latter decades of our study (Figure 2b). Considering only significant changes in abundance, we find that species increasing in abundance show less of a decline in synchrony during the first two decades of our study (Figure S9). This is consistent with positive density‐dependent emigration, whereby higher population density due to increasing population abundance can facilitate the spread of individuals (Hanski 1998; Roland et al. 2000). This offers a potential avenue for in situ conservation to enable connectivity between habitat patches (Hodgson et al. 2011). However, between 2000 and 2012, species significantly declining in abundance show a stronger increase in synchrony during the latter two decades of our study compared to those increasing in abundance (Figure S10). This suggests a potentially complex relationship between population synchrony with abundance. For example, although higher abundance at the site level (linked to habitat quality and extent) could induce emigration and promote synchrony (Matter et al. 2003), it may be that populations at the edge of their geographic range are declining in abundance as is often observed (Guo et al. 2005), combined with these populations being more climatically constrained and having fewer suitable microclimates available resulting in synchronised extinctions and re‐colonisations (Powney et al. 2010).

Overall, after accounting for synchrony in temperature and rainfall, our results are consistent with the view that changes in population synchrony are influenced by mobility‐related attributes (Table 1). However, the strength of density regulation between populations can affect how well dispersal contributes to population synchrony (Hansen et al. 2020; Lande et al. 1999). If populations are weakly regulated by density, dispersal can increase the scale of population synchrony even if individual dispersal is low (Lande et al. 1999). Hence understanding the strength of density dependence can help further examine the role of dispersal in synchronising population dynamics. Furthermore, although we account for the direct impact of climate on population synchrony, climate could be indirectly driving synchronised population dynamics by altering dispersal rates, or by influencing habitat fragmentation, which can disrupt dispersal patterns (Hansen et al. 2020). It should also be considered that a number of expected patterns showed non‐significant results, which could be due to low statistical power where a large number of interacting factors affect population dynamics causing lower effect sizes. Note also, when assessing temporal trends in population synchrony, we assumed monotonous trends given the complexity of our statistical model (Appendix S2), whereby the influence of a species attribute on change in population synchrony over time is assessed through the interaction effect between species attribute and year in our ‘all species model’. Allowing for non‐linear effects would require including quadratic effects for year and the interaction terms. We felt the time span in our dataset yielded insufficient statistical power to test hypotheses related to quadratic term interaction effects, and the interpretation in a multi‐species model would be very complex. However, there is some evidence on non‐linearity of trends in population synchrony over time for certain species (Figures S2 and S3), and this could be explored in further work, preferably using single‐species models to avoid issues with statistical power.

We found a greater number of significant results overall for butterflies compared to birds. This could be explained by CBC having many fewer sites in total and only 8–10 visits per year, and BBS having only two site visits per year with over three times the number of sites compared to UKBMS sites. Fewer site visits per year lead to higher uncertainty around annual indices of population size, making patterns of local abundance and synchrony much harder to detect. Although we find several significant results, our effect sizes (and *r* ^2^ values) are low. This could be due to some climate variables being unaccounted for that are driving species population synchrony patterns. However, seasonal temperature and rainfall have been shown to capture other climate variables; for example, the decline of wren populations with the number of frost nights can also be captured using mean winter temperature (Bellamy et al. 2003). Therefore, using climate variables tailored to species‐specific temporal windows would likely lead to broadly similar results as we have here, though explained variance in synchrony may slightly improve.

In conclusion, our analyses reveal a widespread effect of mobility‐related attributes and abundance patterns on population synchrony over time, after accounting for seasonal temperature and rainfall as a confounding effect. These results suggest that dispersal is a key mechanism contributing to the synchrony of population trends, and that a more complete understanding of population synchrony can only be achieved through considering the role of dispersal in conjunction with additional climatic drivers, including temporal trends in climatic spatial autocorrelation.

## Author Contributions

**Lisbeth A. Hordley:** data curation (equal), formal analysis (lead), investigation (equal), methodology (equal), project administration (equal), writing – original draft (lead), writing – review and editing (equal). **Gary D. Powney:** formal analysis (supporting), methodology (equal), supervision (equal), writing – review and editing (equal). **Tom Brereton:** writing – review and editing (equal). **Simon Gillings:** writing – review and editing (equal). **Owen L. Petchey:** conceptualization (equal), supervision (equal), writing – review and editing (equal). **David B. Roy:** writing – review and editing (equal). **Joseph A. Tobias:** conceptualization (equal), supervision (equal), writing – review and editing (equal). **James Williams:** writing – review and editing (equal). **Tom H. Oliver:** conceptualization (equal), supervision (equal), writing – review and editing (equal).

## Conflicts of Interest

The authors declare no conflicts of interest.

## Supporting information


Data S1.

## Data Availability

The data supporting the results (population synchrony scores for every pair of sites for every species for each 10‐year window and climate synchrony values for every pair of sites for each 10‐year window) and associated R code are available on GitHub (https://github.com/lhordley/Population‐synchrony).
