# Supplementary material for: Disentangling How Climate and Dispersal Drive Temporal Trends in Synchronous Population Dynamics
Source: Ecol Evol. 2025 May 26;15(5):e71443. doi: 10.1002/ece3.71443 (PMC12105940; doi:10.1002/ece3.71443)
Supplement: Supplementary file 1 — Data S1. [file ECE3-15-e71443-s001.docx]

**Disentangling how climate and dispersal drive temporal trends in synchronous population dynamics**

**Supplementary Material Appendix**

Lisbeth A Hordley^a,b,^*, Gary D Powney^c^, Tom Brereton^b^, Simon Gillings^d^, Owen L Petchey^e^, David B Roy^c^, Joseph A Tobias^f^, James Williams^g^, and Tom H Oliver^a^

*Corresponding author: [lhordley@butterfly-conservation.org](mailto:lhordley@butterfly-conservation.org)


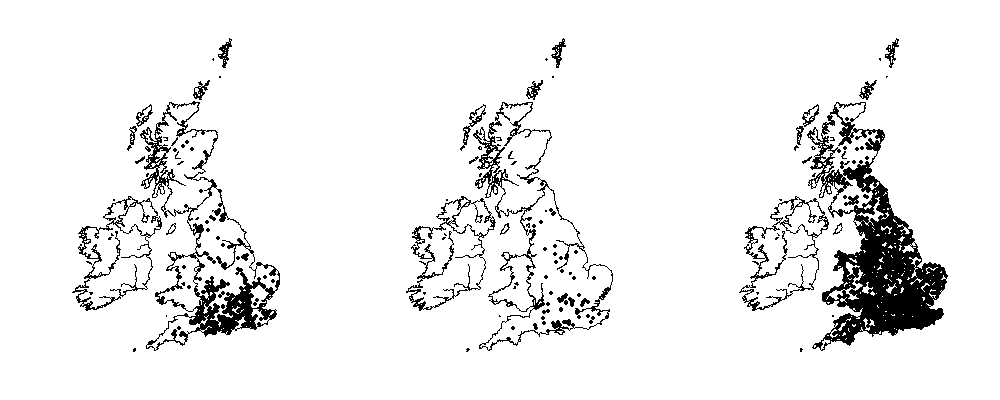


**(a) (b) (c)**

**Figure A1.** Maps of the UK with points showing locations for all sites included in the analysis for (a) UKBMS, (b) CBC, and (c) BBS schemes with a total of 686, 106 and 2490 sites respectively.


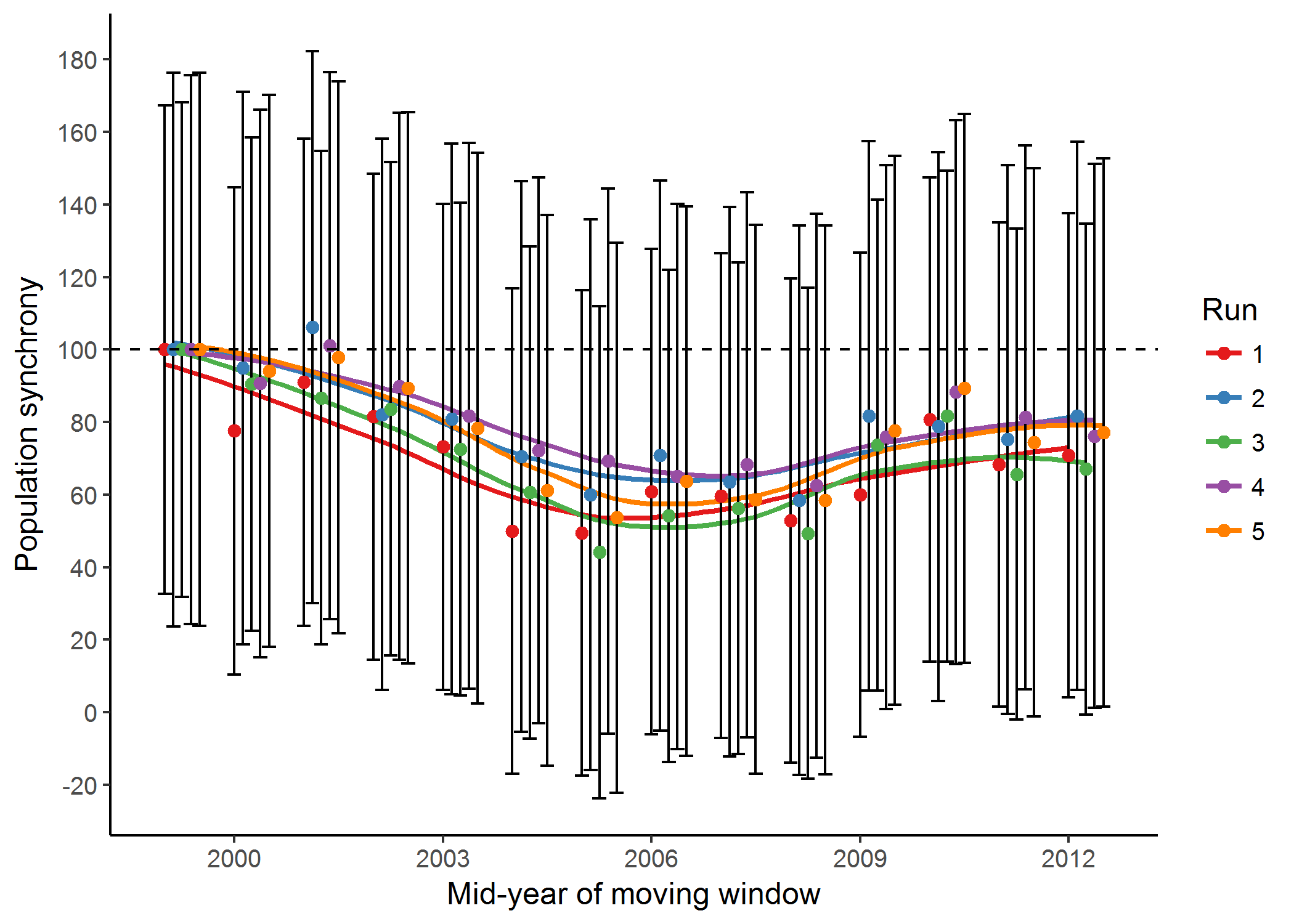


**Figure A2.** The temporal trend in residual population synchrony fitted using a LOESS regression function with standard error bars for BBS birds showing the five repeated runs to calculate population synchrony using a different random subset of 10,000 sites when species had large amounts of data. The first year (1999) population synchrony value for each run is set to a value of 100.


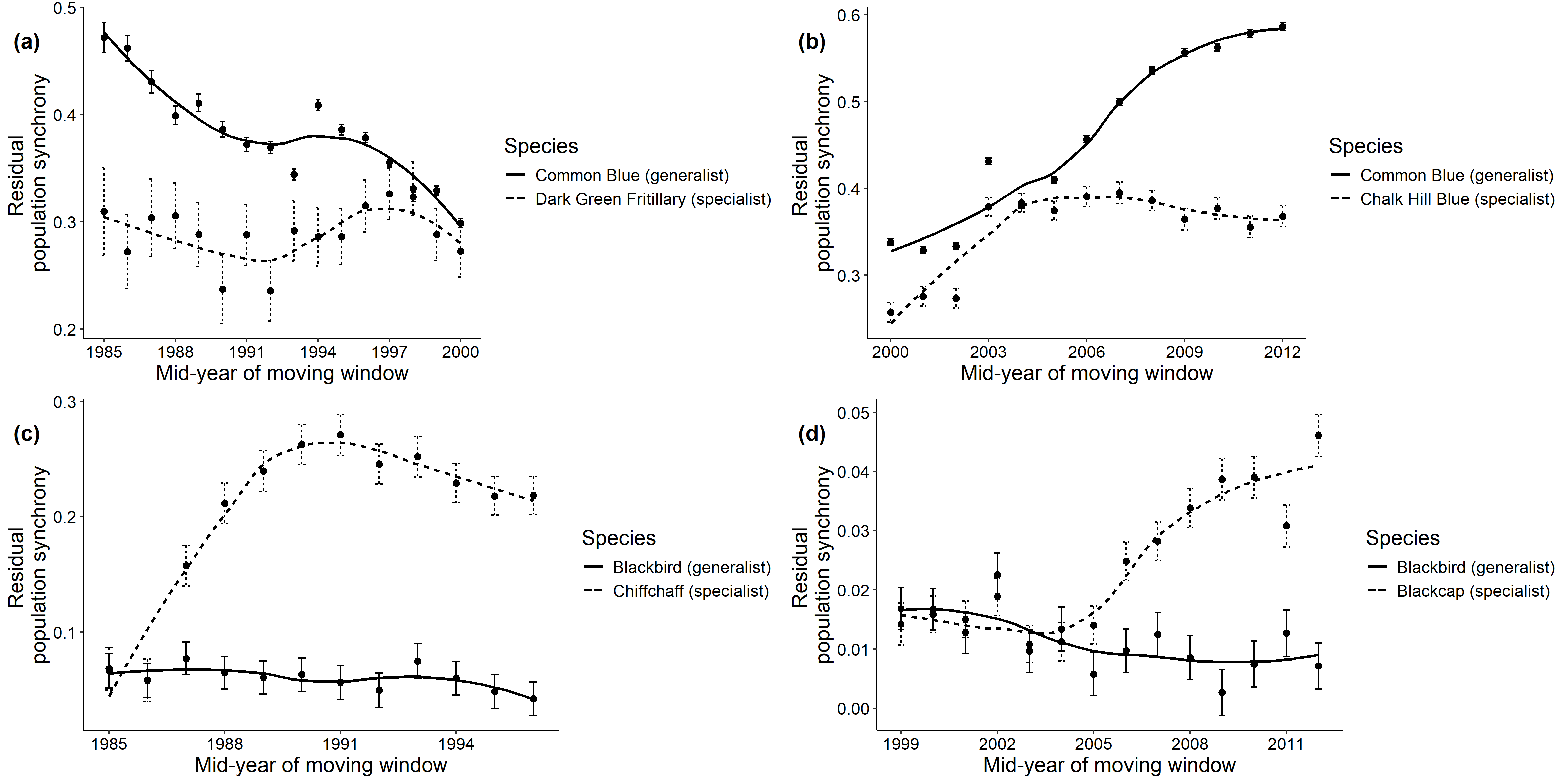


**Figure A3.** Temporal trends in population synchrony for butterflies between (a) 1985 and 2000 and (b) 2000 and 2012, and for (c) CBC birds between 1985 and 1996 and (d) BBS birds between 1999 and 2012. Species represented here were selected to demonstrate the different temporal trends in population synchrony for specialist and generalist species. For UKBMS species, generalists showed the greatest decline in synchrony in the first two decades, followed by the greatest increase in the latter two decades. For both BBS and CBC birds, specialists showed greater increases in population synchrony over time compared to generalists. The temporal trend in population synchrony was fitted using a LOESS regression function with standard error bars around each residual population synchrony value.


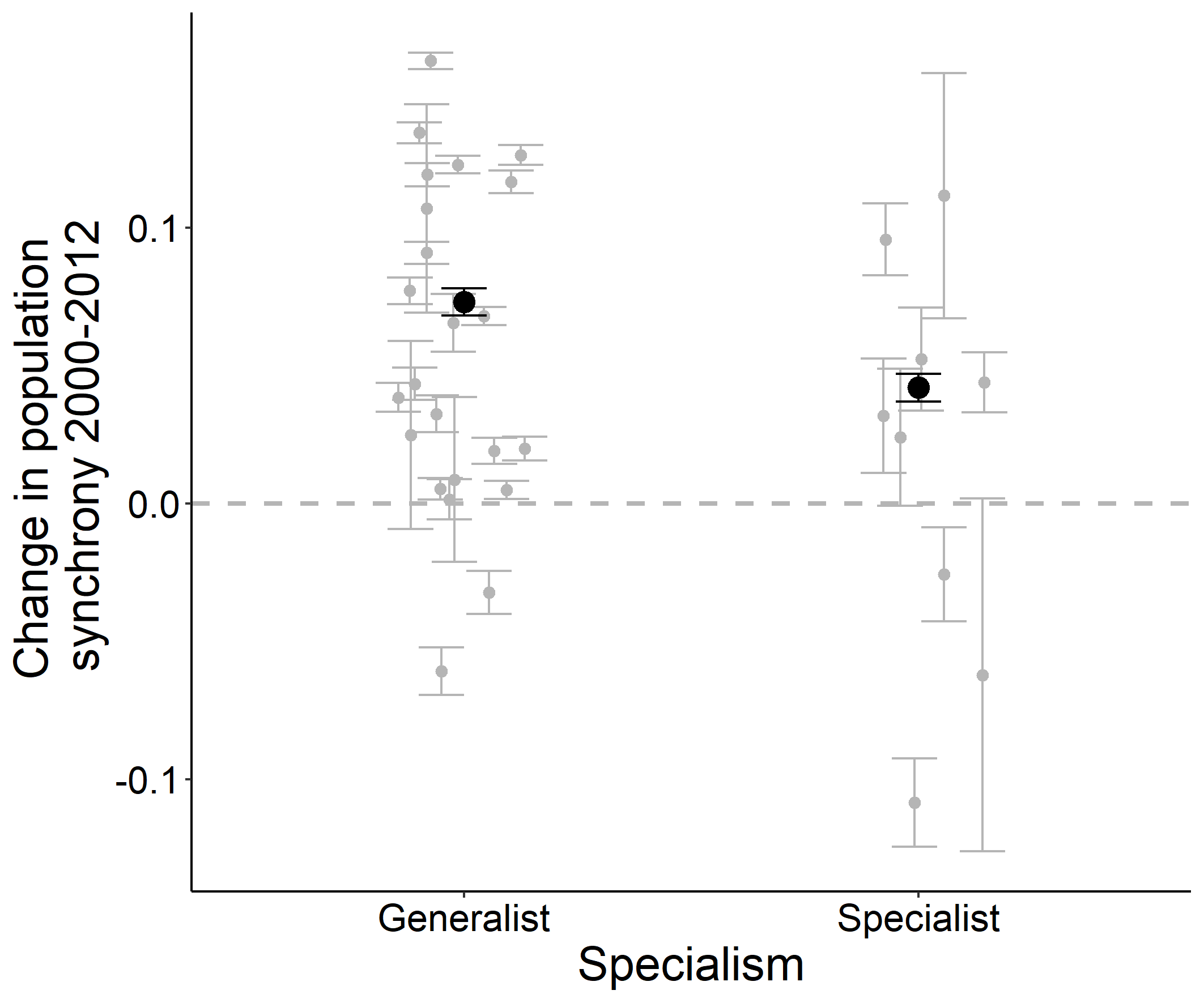
**Figure A4.** Change in population synchrony over time for UKBMS butterflies in relation to specialism between 2000 and 2012. The dashed grey line represents zero change in population synchrony over time, grey points represent each species raw data with standard error bars, and black points represent the slope (i.e. change in synchrony over time) from the mixed effects models with their associated standard errors. Grey points were scattered horizontally randomly with a small deviation to increase clarity.


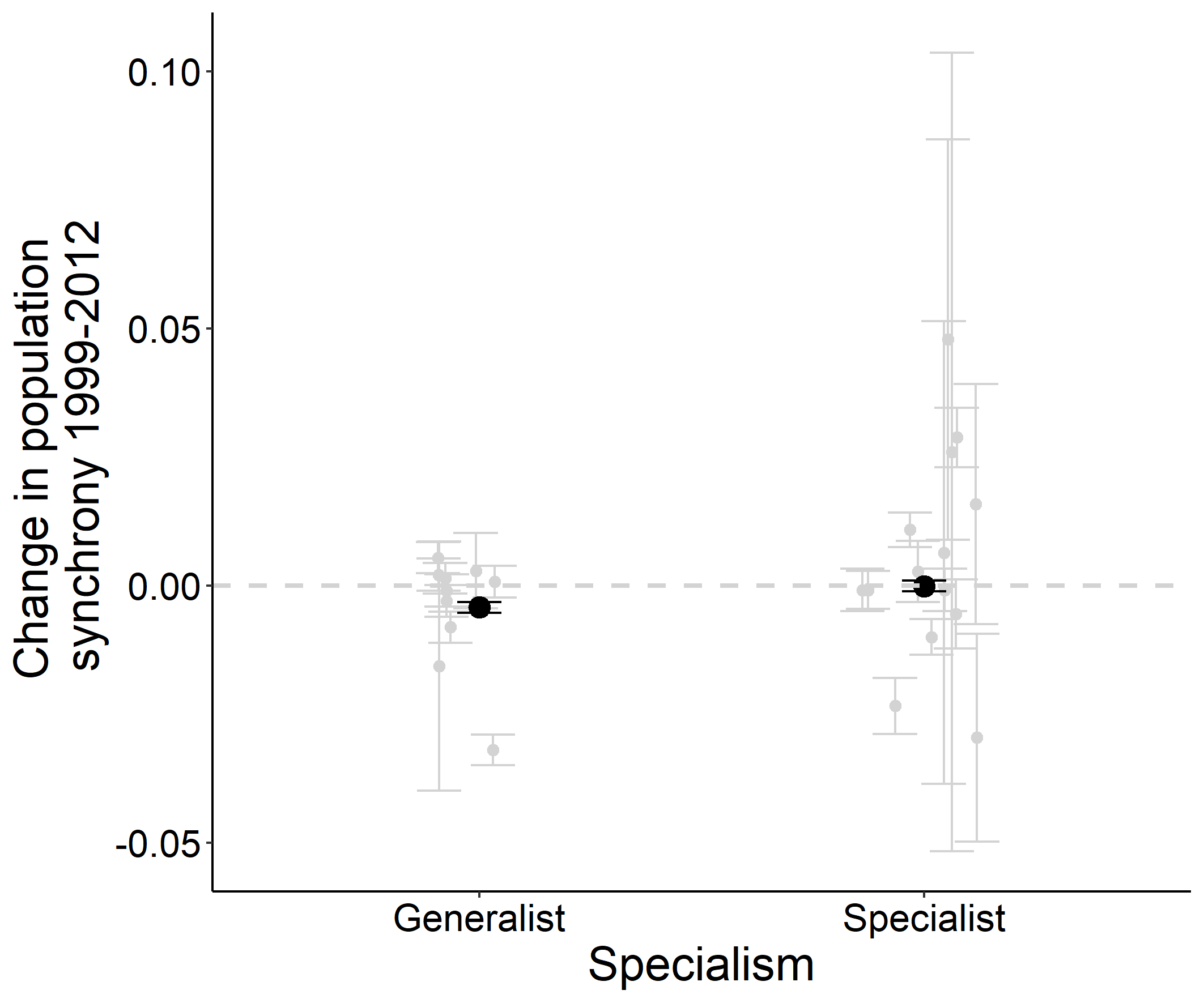
**Figure A5.** Change in population synchrony over time for BBS birds in relation to specialism between 1999 and 2012. Dashed grey lines represent zero change in population synchrony over time, grey points represent each species raw data with standard error bars, and black points represent the slope (i.e. change in synchrony over time) from the mixed effects models with their associated standard errors. Grey points were scattered horizontally randomly with a small deviation to increase clarity.

**
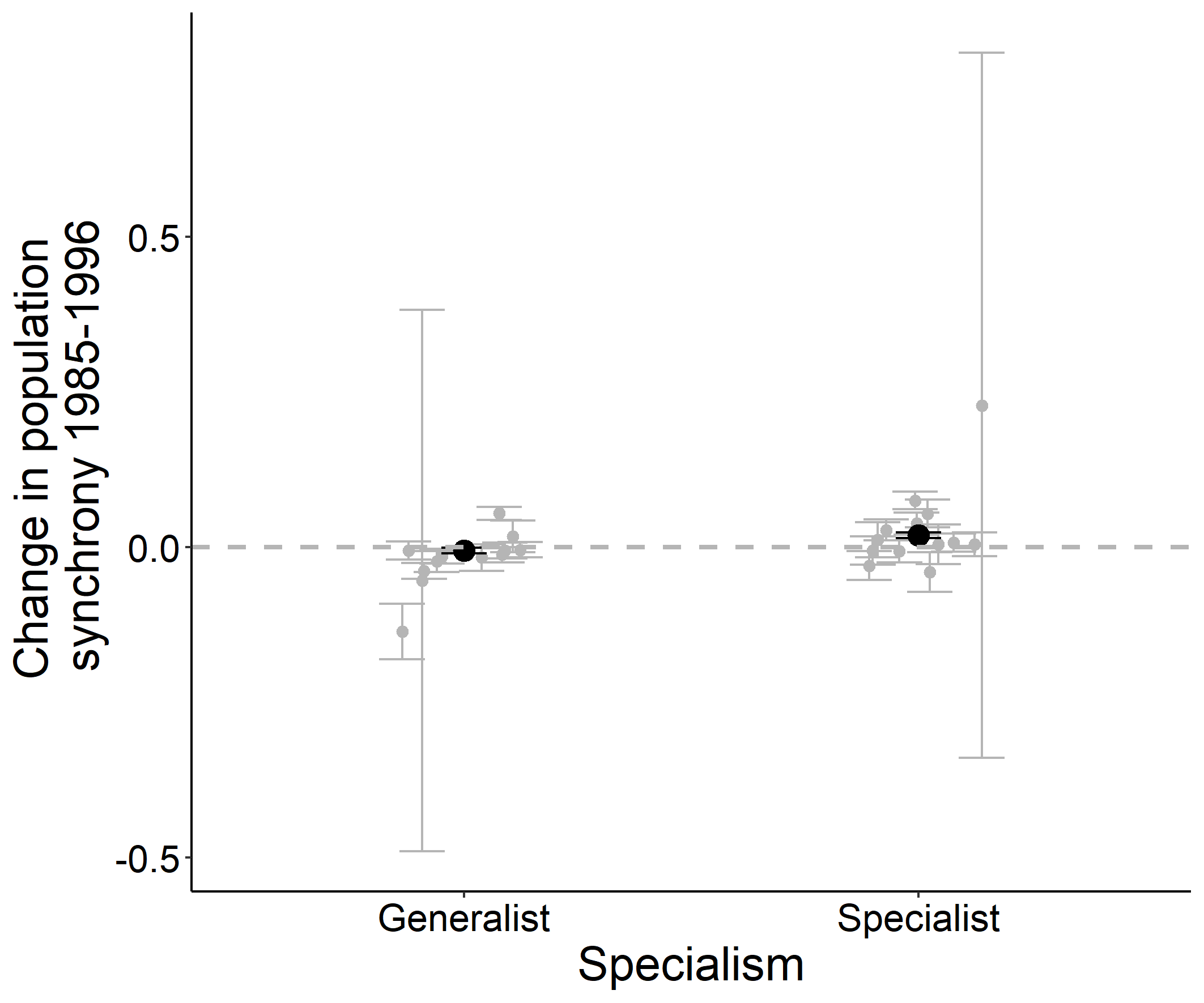
Figure A6.** Change in population synchrony over time for CBC birds in relation to specialism between 1985 and 1996. Dashed grey lines represent zero change in population synchrony over time, grey points represent each species raw data with standard error bars, and black points represent the slope (i.e. change in synchrony over time) from the mixed effects models with their associated standard errors. Grey points were scattered horizontally randomly with a small deviation to increase clarity.

**
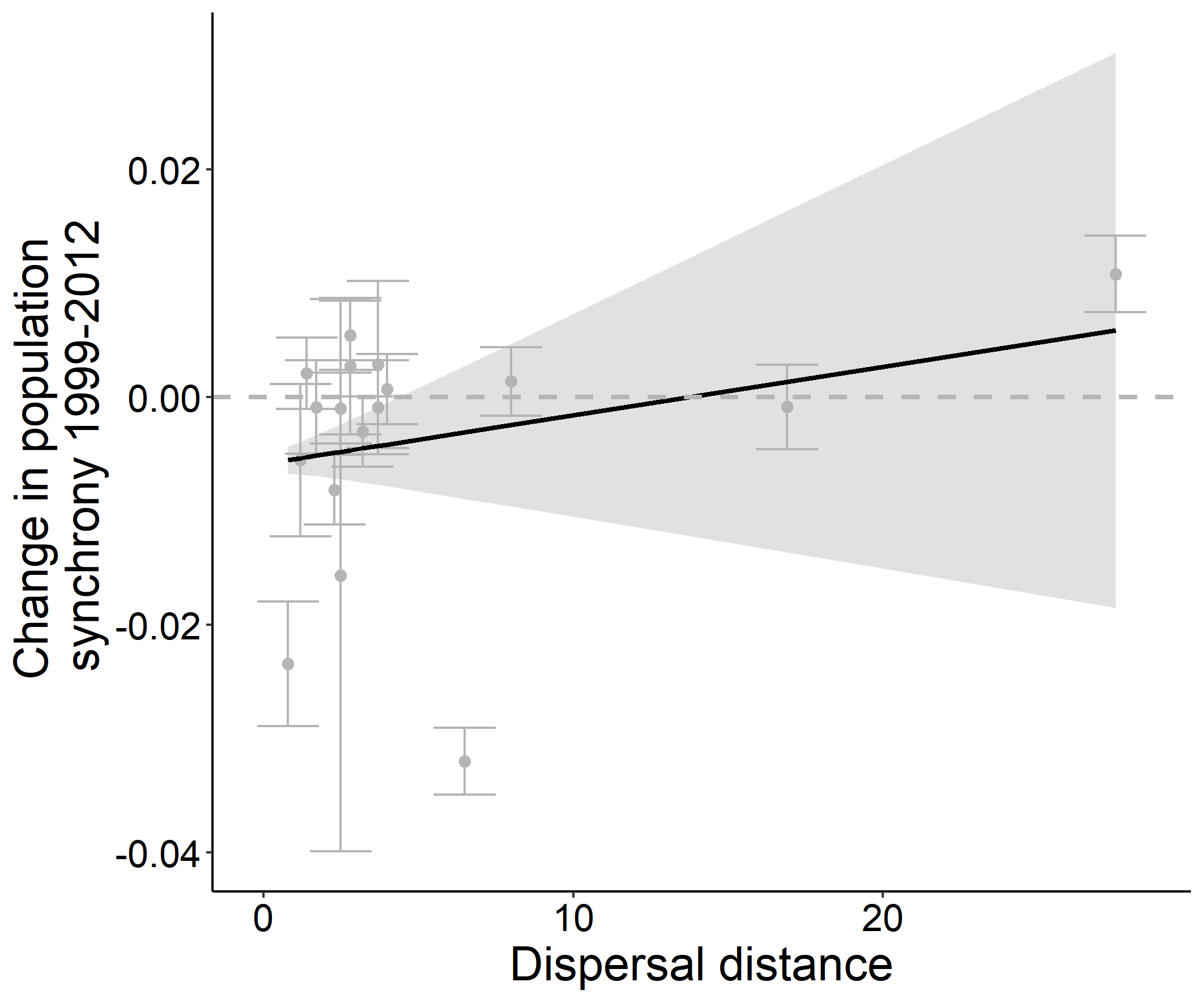
Figure A7**. The change in population synchrony over time for BBS birds in relation to mobility between 1999 and 2012. The dashed grey line represents zero change in population synchrony over time, grey points represent each species raw data with standard error bars, and the solid line represents the slope (i.e. change in synchrony over time) for each mobility score from the mixed effects models with the associated standard error. Grey points were scattered horizontally randomly with a small deviation to increase clarity.

**
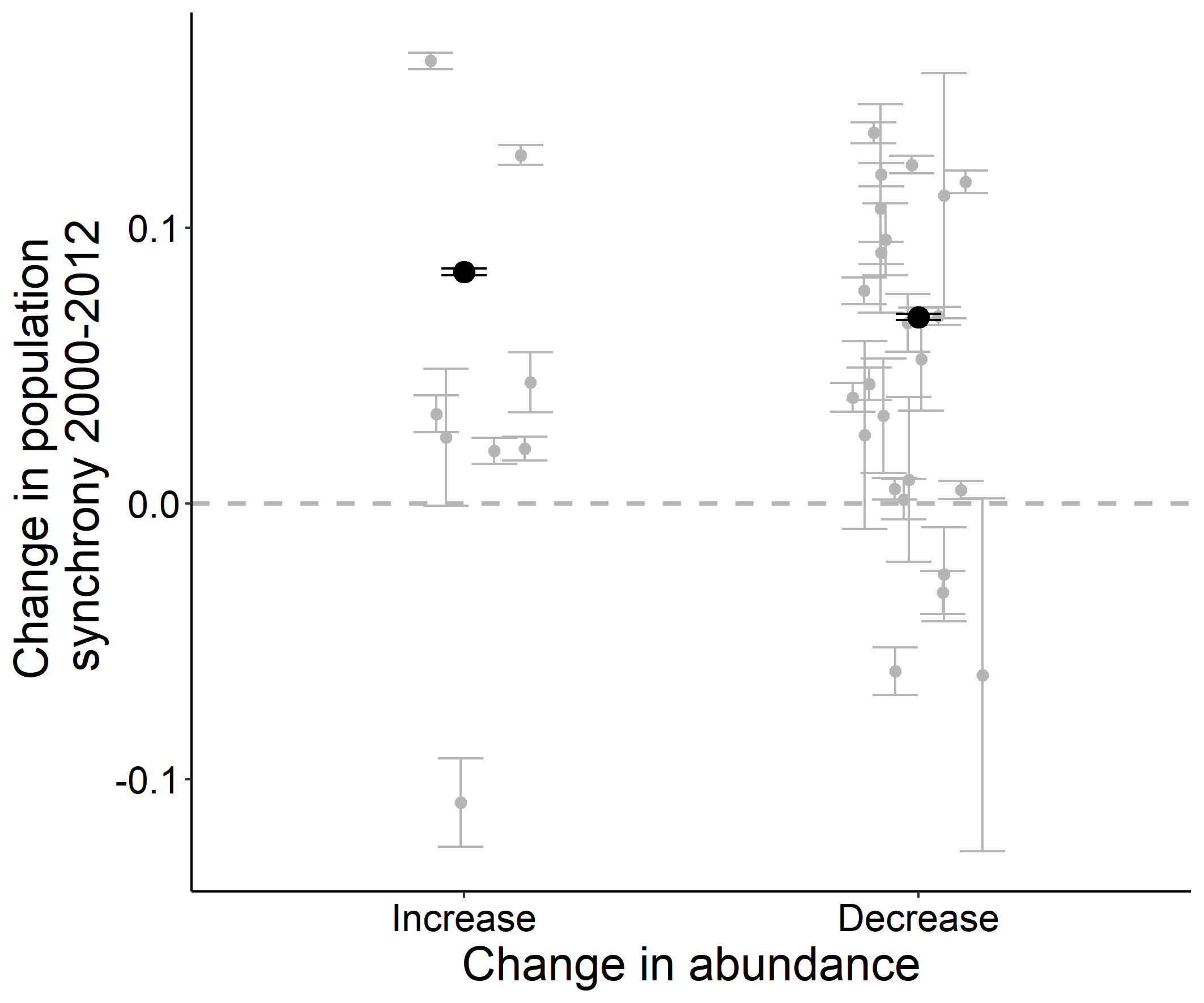
Figure A8**. The change in population synchrony over time for UKBMS butterflies in relation to non-significant changes in abundance between 2000 and 2012. The dashed grey line represents zero change in population synchrony over time, grey points represent each species raw data with standard error bars, and black points represent the slope (i.e. change in synchrony over time) from the mixed effects models with their associated standard errors. Grey points were scattered horizontally randomly with a small deviation to increase clarity.


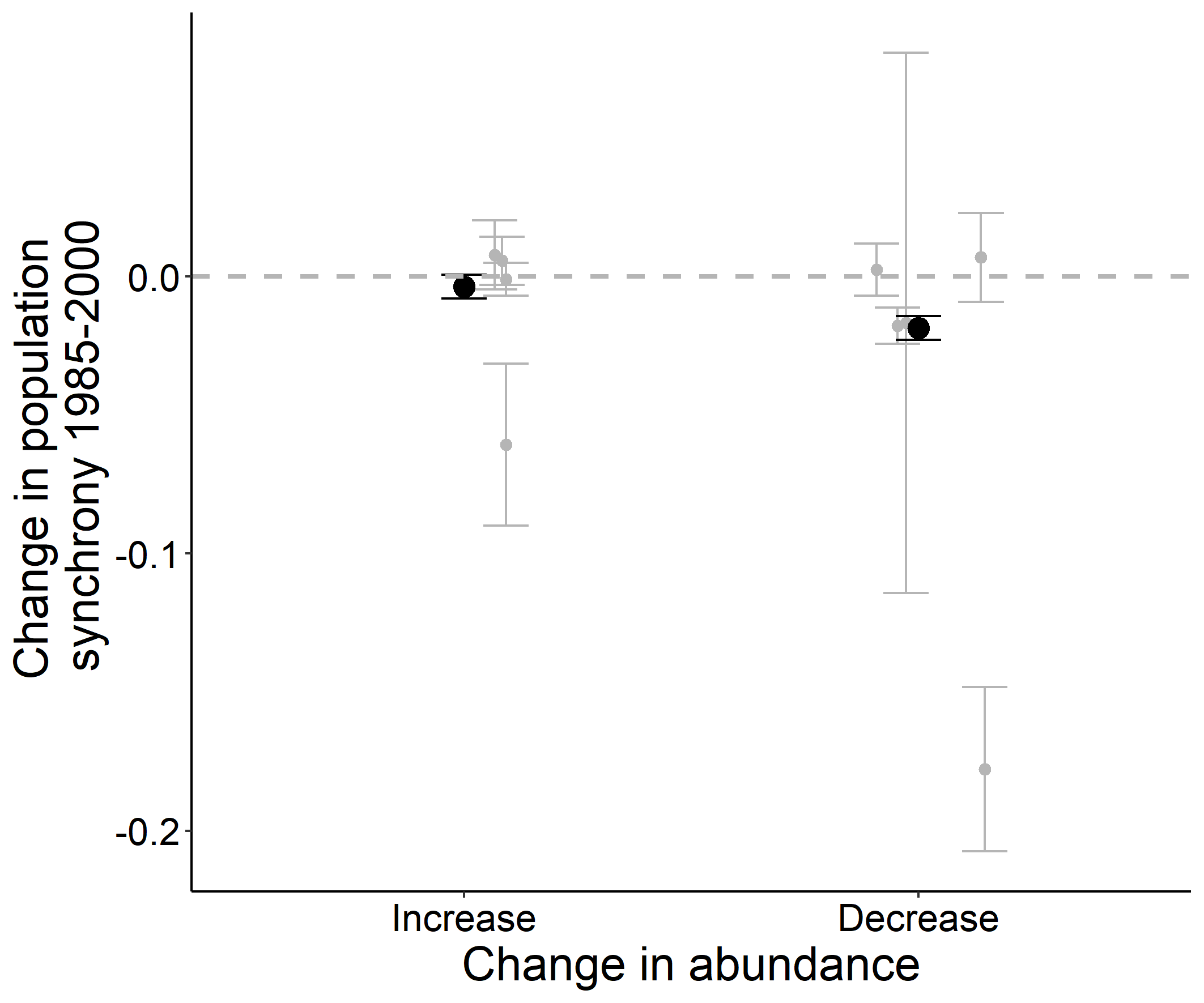
**Figure A9**. The change in population synchrony over time for UKBMS butterflies in relation to significant changes in abundance between 1985 and 2000. The dashed grey line represents zero change in population synchrony over time, grey points represent each species raw data with standard error bars, and black points represent the slope (i.e. change in synchrony over time) from the mixed effects models with their associated standard errors. Grey points were scattered horizontally randomly with a small deviation to increase clarity.


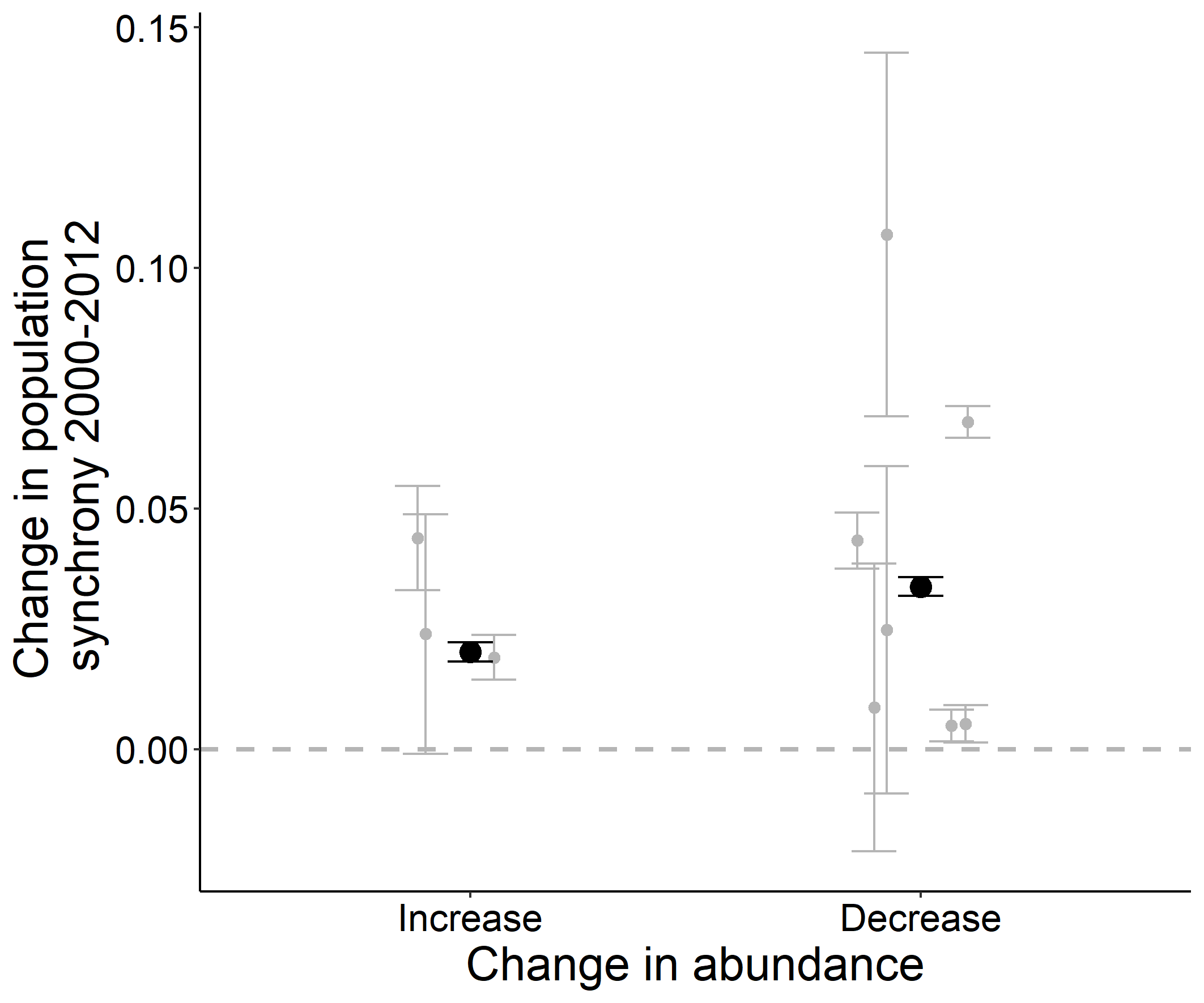
**Figure A10**. The change in population synchrony over time for UKBMS butterflies in relation to significant changes in abundance between 2000 and 2012. The dashed grey line represents zero change in population synchrony over time, grey points represent each species raw data with standard error bars, and black points represent the slope (i.e. change in synchrony over time) from the mixed effects models with their associated standard errors. Grey points were scattered horizontally randomly with a small deviation to increase clarity.

**Table A1.** Summary data of each butterfly species used in the analysis including common and Latin name, biotype specialism, relative mobility, average abundance, abundance change, and change in synchrony over time. Dashes represent missing data or species with insufficient data. See main text for source data on species attributes.

| **Common name** | **Latin name** | **Biotype Specialism** | **Mobility rank** | **Average abundance between 2009-2016** | **Abundance change 85-00** | **Abundance change 00-12** | **Synchrony change 85-00** | **Synchrony change 00-12** |
| --- | --- | --- | --- | --- | --- | --- | --- | --- |
| Brimstone | *Gonepteryx rhamni* | Generalist | 46 | 279 | Increase | Decrease | Decrease | Increase |
| Brown argus | *Aricia agestis* | Generalist | 14 | 140 | Increase | Decrease | Decrease | Increase |
| Chalk-hill blue | *Polyommatus coridon* | Specialist | 13 | 65 | Increase | Decrease | No change | Increase |
| Comma | *Polygonia c-album* | Generalist | 42 | 680 | Increase | Increase | No change | Increase |
| Common blue | *Polyommatus icarus* | Generalist | 28 | 1235 | Increase | Decrease | Decrease | Increase |
| Dark green fritillary | *Argynnis aglaja* | Specialist | 37 | 58 | Increase | Increase | Decrease | No change |
| Dingy skipper | *Erynnis tages* | Specialist | 10 | 6 | Decrease | Increase | No change | Decrease |
| Essex skipper | *Thymelicus lineola* | Generalist | 23 | 286 | Decrease | Decrease | − | No change |
| Gatekeeper | *Pyronia tithonus* | Generalist | 28 | 7168 | Increase | Decrease | Decrease | Increase |
| Grayling | *Hipparchia semele* | Specialist | 29 | 164 | Decrease | Decrease | − | Increase |
| Green hairstreak | *Callophrys rubi* | Specialist | 17 | 4 | Decrease | Decrease | − | Increase |
| Green-veined white | *Pieris napi* | Generalist | 45 | 5153 | Decrease | Decrease | Increase | Increase |
| Grizzled skipper | *Pyrgus malvae* | Specialist | 8 | − | Increase | Decrease | − | No change |
| Holly blue | *Celastrina argiolus* | Generalist | 44 | 245 | Increase | Decrease | − | Decrease |
| Large skipper | *Ochlodes sylvanus* | Generalist | 27 | 612 | Decrease | Decrease | Decrease | Increase |
| Large white | *Pieris brassicae* | Generalist | 57 | 6854 | Decrease | Increase | Decrease | Increase |
| **Common name** | **Latin name** | **Biotype Specialism** | **Mobility rank** | **Average abundance between 2009-2016** | **Abundance change 85-00** | **Abundance change 00-12** | **Synchrony change 85-00** | **Synchrony change 00-12** |
| Marbled white | *Melanargia galathea* | Generalist | 31 | 739 | Increase | Decrease | Decrease | Increase |
| Meadow brown | *Maniola jurtina* | Generalist | 32 | 15339 | Increase | Decrease | Decrease | Increase |
| Orange tip | *Anthocharis cardamines* | Generalist | 41 | 17 | Decrease | Increase | Decrease | Increase |
| Peacock | *Aglais io* | Generalist | 54 | 1942 | Increase | Decrease | Decrease | Increase |
| Purple hairstreak | *Favonius quercus* | Specialist | 18 | 57 | Increase | Decrease | − | No change |
| Ringlet | *Aphantopus hyperantus* | Generalist | 22 | 6600 | Increase | Increase | No change | Increase |
| Silver-washed fritillary | *Argynnis paphia* | Specialist | 39 | 203 | Increase | Increase | − | Increase |
| Small copper | *Lycaena phlaeas* | Generalist | 33 | 338 | Decrease | Decrease | Decrease | Increase |
| Small heath | *Coenonympha pamphilus* | Generalist | 23 | 673 | Decrease | Decrease | No change | Decrease |
| Small pearl-bordered fritillary | *Boloria selene* | Specialist | 25 | 37 | Decrease | Decrease | − | − |
| Small skipper | *Thymelicus sylvestris* | Generalist | 25 | 1116 | Decrease | Decrease | Decrease | Increase |
| Small tortoiseshell | *Aglais urticae* | Generalist | 51 | 3270 | Decrease | Decrease | Decrease | Increase |
| Small white | *Pieris rapae* | Generalist | − | 9696 | Increase | Decrease | Decrease | Increase |
| Speckled wood | *Pararge aegeria* | Generalist | 30 | 2526 | Increase | Increase | Decrease | Increase |
| Wall brown | *Lasiommata megera* | Generalist | 38 | 207 | Decrease | Decrease | Decrease | No change |
| White admiral | *Limenitis camilla* | Specialist | 34 | 43 | Decrease | Decrease | No change | Increase |

**Table A2.** Summary of all CBC bird species used in the analysis including common and Latin names, biotype specialism, relative mobility, average abundance, abundance change, and change in synchrony over time. Dashes represent missing data or species with insufficient data. See main text for source data on species attributes.

| **Common name** | **Latin name** | **Biotype Specialism** | **Dispersal distance (km)** | **Average abundance (number of pairs/territories)** | **Abundance change** | **Synchrony change** |
| --- | --- | --- | --- | --- | --- | --- |
| Blackbird | *Turdus merula* | Generalist | 3.2 | 4900000 | Decrease | No change |
| Blackcap | *Sylvia atricapilla* | Specialist | 27.5 | 1100000 | Increase | No change |
| Blue tit | *Cyanistes caeruleus* | Generalist | 2.3 | 3400000 | Increase | No change |
| Bullfinch | *Pyrrhula pyrrhula* | Generalist | 2.5 | 190000 | Decrease | No change |
| Chaffinch | *Fringilla coelebs* | Generalist | 2.8 | 5800000 | Increase | No change |
| Chiffchaff | *Phylloscopus collybita* | Specialist | − | 1100000 | Increase | Increase |
| Coal tit | *Periparus ater* | Specialist | 1.2 | 680000 | Increase | No change |
| Dunnock | *Prunella modularis* | Generalist | 1.4 | 2300000 | Decrease | No change |
| Garden warbler | *Sylvia borin* | Specialist | − | 170000 | Increase | No change |
| Goldcrest | *Regulus regulus* | Specialist | − | 520000 | − | No change |
| Great spotted woodpecker | *Dendrocopos major* | Specialist | 3.7 | 140000 | Increase | No change |
| Great tit | *Parus major* | Generalist | 2.5 | 2500000 | Increase | No change |
| Green woodpecker | *Picus viridis* | Specialist | 1.7 | 52000 | − | No change |
| Jay | *Garrulus glandarius* | Specialist | 2.8 | 4300 | Decrease | Increase |
| Lesser whitethroat | *Sylvia curruca* | Generalist | 16.4 | 74000 | Decrease | − |
| Long-tailed tit | *Aegithalos caudatus* | Generalist | 3.7 | 330000 | − | No change |
| Marsh tit | *Poecile palustris* | Specialist | − | 41000 | Decrease | No change |
| Nuthatch | *Sitta europaea* | Specialist | 0.8 | 220000 | Increase | Increase |
| Redstart | *Phoenicurus phoenicurus* | Specialist | − | 100000 | Increase | − |
| Robin | *Erithacus rubecula* | Generalist | 8 | 6000000 | Increase | Decrease |
| Song thrush | *Turdus philomelos* | Generalist | 4 | 1100000 | Decrease | No change |
| **Common name** | **Latin name** | **Biotype Specialism** | **Dispersal distance (km)** | **Average abundance (number of pairs/territories)** | **Abundance change** | **Synchrony change** |
| Tawny owl | *Strix aluco* | Generalist | 3.1 | 50000 | Decrease | Decrease |
| Treecreeper | *Certhia familiaris* | Specialist | − | 180000 | Decrease | No change |
| Willow tit | *Poecile montanus* | Specialist | 1.7 | 3400 | Decrease | − |
| Willow warbler | *Phylloscopus trochilus* | Specialist | 16.9 | 2200000 | − | No change |
| Wren | *Troglodytes troglodytes* | Generalist | 6.5 | 7700000 | Increase | Increase |

**Table A3.** Summary of all BBS bird species used in the analysis including common and Latin names, biotype specialism, relative mobility, average abundance, abundance change, and change in synchrony over time. Dashes represent missing data or species with insufficient data. See main text for source data on species attributes.

| **Common name** | **Latin name** | **Biotype Specialism** | **Dispersal distance (km)** | **Average abundance (number of pairs/territories)** | **Abundance change** | **Synchrony change** |
| --- | --- | --- | --- | --- | --- | --- |
| Blackbird | *Turdus merula* | Generalist | 3.2 | 4900000 | Increase | No change |
| Blackcap | *Sylvia atricapilla* | Specialist | 27.5 | 1100000 | Increase | Increase |
| Blue tit | *Cyanistes caeruleus* | Generalist | 2.3 | 3400000 | Increase | Decrease |
| Bullfinch | *Pyrrhula pyrrhula* | Generalist | 2.5 | 190000 | Increase | No change |
| Chaffinch | *Fringilla coelebs* | Generalist | 2.8 | 5800000 | Increase | No change |
| Chiffchaff | *Phylloscopus collybita* | Specialist | − | 1100000 | Increase | Decrease |
| Coal tit | *Periparus ater* | Specialist | 1.2 | 680000 | Increase | No change |
| Dunnock | *Prunella modularis* | Generalist | 1.4 | 2300000 | Increase | No change |
| Garden warbler | *Sylvia borin* | Specialist | − | 170000 | Decrease | No change |
| Goldcrest | *Regulus regulus* | Specialist | − | 520000 | − | Increase |
| Great spotted woodpecker | *Dendrocopos major* | Specialist | 3.7 | 140000 | Increase | No change |
| Great tit | *Parus major* | Generalist | 2.5 | 2500000 | Increase | No change |
| Green woodpecker | *Picus viridis* | Specialist | 1.7 | 52000 | − | No change |
| Jay | *Garrulus glandarius* | Specialist | 2.8 | 4300 | Increase | No change |
| Lesser redpoll | *Carduelis cabaret* | Specialist | − | 190000 | − | No change |
| Long-tailed tit | *Aegithalos caudatus* | Generalist | 3.7 | 330000 | − | No change |
| Nuthatch | *Sitta europaea* | Specialist | 0.8 | 220000 | Increase | Decrease |
| Redstart | *Phoenicurus phoenicurus* | Specialist | − | 100000 | Increase | No change |
| Robin | *Erithacus rubecula* | Generalist | 8 | 6000000 | Increase | No change |
| Song thrush | *Turdus philomelos* | Generalist | 4 | 1100000 | Increase | No change |
| **Common name** | **Latin name** | **Biotype Specialism** | **Dispersal distance (km)** | **Average abundance (number of pairs/territories)** | **Abundance change** | **Synchrony change** |
| Tree pipit | *Anthus trivialis* | Specialist | − | 88000 | − | No change |
| Treecreeper | *Certhia familiaris* | Specialist | − | 180000 | Increase | − |
| Willow warbler | *Phylloscopus trochilus* | Specialist | 16.9 | 2200000 | − | No change |
| Wren | *Troglodytes troglodytes* | Generalist | 6.5 | 7700000 | Decrease | Decrease |

**Table A4.** Results from linear mixed models for each dataset with population synchrony as the response variable and climate synchrony as explanatory variables. Significant climate variables (p<0.05) were included as covariates in future analyses.

| **Dataset** | | **Climate synchrony variable** | **Estimate** | **t-value** | **SE** | **p-value** |
| --- | --- | --- | --- | --- | --- | --- |
| UKBMS | | Spring temperature | 0.019 | 43.21 | 0.0005 | <0.001*** |
|  |  | Summer temperature | 0.0003 | 0.63 | 0.0005 | 0.53 |
|  |  | Autumn temperature | 0.01 | 20.4 | 0.0005 | <0.001*** |
|  |  | Winter temperature | 0.015 | 27.58 | 0.0005 | <0.001*** |
|  |  | Spring rainfall | 0.014 | 34.03 | 0.0004 | <0.001*** |
|  |  | Summer rainfall | 0.0035 | 7.95 | 0.0004 | <0.001*** |
|  |  | Autumn rainfall | -0.0013 | -2.77 | 0.0005 | 0.006** |
|  |  | Winter rainfall | 0.0023 | 4.68 | 0.0005 | <0.001*** |
| CBC | | Spring temperature | 0.0054 | 1.80 | 0.003 | 0.072 |
|  |  | Summer temperature | 0.0067 | 2.06 | 0.0003 | 0.040* |
|  |  | Autumn temperature | -0.0014 | -0.67 | 0.002 | 0.50 |
|  |  | Winter temperature | -0.0049 | -1.88 | 0.003 | 0.061 |
|  |  | Spring rainfall | -0.0021 | -0.83 | 0.003 | 0.41 |
|  |  | Summer rainfall | 0.0038 | 1.31 | 0.003 | 0.19 |
|  |  | Autumn rainfall | 0.0032 | 1.22 | 0.003 | 0.22 |
|  |  | Winter rainfall | -0.00086 | -0.33 | 0.003 | 0.74 |
| BBS | | Spring temperature | -0.00011 | -0.25 | 0.0003 | 0.80 |
|  |  | Summer temperature | -0.00025 | -0.53 | 0.0005 | 0.60 |
|  |  | Autumn temperature | 0.0013 | 2.95 | 0.0004 | 0.0032** |
|  |  | Winter temperature | -0.00016 | -0.34 | 0.0005 | 0.73 |
|  |  | Spring rainfall | 0.0011 | 2.89 | 0.0004 | 0.0039** |
|  |  | Summer rainfall | -0.00041 | -0.80 | 0.0005 | 0.42 |
|  |  | Autumn rainfall | 0.00039 | 0.80 | 0.0005 | 0.42 |
|  |  | Winter rainfall | 0.0014 | 3.22 | 0.0004 | 0.0013** |
|  | **Significance is as follows: ***p < .001, **p < .01, *p < .05.** | | | | | |

**Table A5.** Results from randomisation tests with 1,000 permutations to determine the significance of individual climate synchrony predictor variables where mixed effect models produced significant results (<0.05).

| **Dataset** | | **Climate synchrony variable** | **F value** | **p-value** |
| --- | --- | --- | --- | --- |
| UKBMS | | Spring temperature | 21.93 | 0*** |
|  |  | Autumn temperature | 416.13 | 0*** |
|  |  | Winter temperature | 760.77 | 0*** |
|  |  | Spring rainfall | 1158.01 | 0*** |
|  |  | Summer rainfall | 63.2 | 0*** |
|  |  | Autumn rainfall | 7.68 | 0.006** |
|  |  | Winter rainfall | 21.93 | 0*** |
| CBC | | Summer temperature | 4.23 | 0.028* |
| BBS | | Autumn temperature | 8.72 | 0.002** |
|  |  | Spring rainfall | 8.35 | 0.003** |
|  |  | Winter rainfall | 10.34 | 0.001** |
| **Significance is as follows: ***p < .001, **p < .01, *p < .05.** | | | | |
|  |  |  |  |  |

**Table A6.** Results from linear mixed effects models for butterflies using species attributes and control variables as explanatory variables with either average synchrony, or change in synchrony over two time periods, as the response variable.

| **Explanatory variable** | | **Response variable** | **No. of species** | **Estimate** | **t value** | **SE** | **p-value** | **Direction of change** |
| --- | --- | --- | --- | --- | --- | --- | --- | --- |
| **Species attributes** | | | | | | | | |
| Biotype specialism | | Average synchrony | 32 | 0.043 | 1.02 | 0.04 | 0.31 | Non-significant |
|  |  | Change in synchrony 1985-2000 |  | -0.021 | -3.87 | 0.005 | <0.001** | Generalists decline in synchrony more steeply compared to specialists |
|  |  | Change in synchrony 2000-2012 |  | 0.031 | 6.25 | 0.005 | <0.001** | Generalists increase in synchrony more steeply compared to specialists |
| Mobility | | Average synchrony | 31 | 0.047 | 2.99 | 0.02 | 0.0057** | More mobile species have higher average levels of synchrony |
|  |  | Change in synchrony 1985-2000 |  | -0.00045 | -0.44 | 0.001 | 0.66 | Non-significant |
|  |  | Change in synchrony 2000-2012 |  | 0.018 | 21.59 | 0.0009 | <0.001*** | Species with high mobility show a greater increase in synchrony over time compared to species with low mobility |
| Average abundance | | Average synchrony | 31 | 0.038 | 1.51 | 0.025 | 0.14 | Non-significant |
| Non-significant changes in abundance | | Change in synchrony 1985-2000 | 32 | -0.0025 | -1.22 | 0.002 | 0.22 | Non-significant |
|  |  | Change in synchrony 2000-2012 |  | 0.016 | 9.10 | 0.002 | <0.001*** | Species increasing in abundance increase in synchrony more rapidly |
| Significant changes in abundance | | Change in synchrony 1985-2000 | 9 | 0.015 | 2.25 | 0.007 | 0.025* | Species decreasing in abundance decline in synchrony more rapidly |
|  |  | Change in synchrony 2000-2012 | 10 | -0.014 | -3.69 | 0.004 | <0.001*** | Species decreasing in abundance increase in synchrony more rapidly |
|  | **Significance is as follows: ***p < .001, **p < .01, *p < .05.** | | | | | | | |

**Table A7.** Results from linear mixed effects models for CBC birds using species attributes and control variables as explanatory variables with either average synchrony, or change in synchrony over two time periods, as the response variable.

| **Explanatory variable** | | **Response variable** | **No. of species** | **Estimate** | **t value** | **SE** | **p-value** | **Direction of change** |
| --- | --- | --- | --- | --- | --- | --- | --- | --- |
| **Species attributes** | | | | | | | | |
| Biotype specialism | | Average synchrony | 26 | -0.067 | -1.79 | 0.038 | 0.09 | Non-significant |
|  |  | Change in synchrony |  | 0.0045 | 3.45 | 0.007 | <0.001*** | Specialists increase in synchrony over time, whereas generalists show a small decrease |
| Mobility | | Average synchrony | 20 | -0.0092 | -0.43 | 0.021 | 0.67 | Non-significant |
|  |  | Change in synchrony |  | 0.00086 | 1.28 | 0.004 | 0.2 | Non-significant |
| Average abundance | | Average synchrony | 26 | 0.057 | 2.73 | 0.021 | 0.013* | Species which are more abundant have higher average levels of synchrony |
| Non-significant changes in abundance | | Change in synchrony | 22 | 0.0027 | 1.88 | 0.008 | 0.06 | Non-significant |
| Significant changes in abundance | | Change in synchrony | 19 | 0.0029 | 1.97 | 0.008 | 0.0492* | Species increasing in abundance increase in synchrony, whereas those declining in abundance show small decline in synchrony |
| Significant changes in abundance (with redstart and lesser whitethroat removed) | |  | 17 | 0.015 | 1.88 | 0.008 | 0.06 | Non-significant |
|  | **Significance is as follows: ***p < .001, **p < .01, *p < .05.** | | | | | | | |

**Table A8.** Results from linear mixed effects models for BBS birds using species attributes and control variables as explanatory variables with either average synchrony or change in synchrony over two time periods as the response variable.

| **Explanatory variable** | | **Response variable** | **No. of species** | **Estimate** | **t value** | **SE** | **p-value** | **Direction of change** |
| --- | --- | --- | --- | --- | --- | --- | --- | --- |
| **Species attributes** | | | | | | | | |
| Biotype specialism | | Average synchrony | 24 | 0.0033 | 0.22 | 0.015 | 0.83 | Non-significant |
|  |  | Change in synchrony |  | 0.0041 | 2.01 | 0.002 | 0.044* | Generalists show a more negative change in synchrony over time |
| Mobility | | Average synchrony | 17 | 0.0063 | 1.10 | 0.006 | 0.29 | Non-significant |
|  |  | Change in synchrony |  | 0.0032 | 3.61 | 0.0009 | <0.001*** | Less mobile species have declined in synchrony over time |
|  |  |  | 13* | 0.00093 | 0.81 | 0.001 | 0.42 | Non-significant |
| Average abundance | | Average synchrony | 24 | 0.01 | 1.34 | 0.007 | 0.20 | Non-significant |
| Non-significant changes in abundance | | Change in synchrony | 18 | -0.04 | -1.12 | 0.036 | 0.26 | Non-significant |
| Significant changes in abundance | | Change in synchrony | 12 | -0.036 | -1.02 | 0.035 | 0.31 | Non-significant |
|  | **Significance is as follows: ***p < .001, **p < .01, *p < .05.**  * Four outlying species with high dispersal distances were removed (blackcap, robin, willow warbler, and wren) | | | | | | | |

**Table A9.** Results from randomisation tests with 1,000 permutations to determine the significance of individual species attribute predictor variables where mixed effect models produced significant results (<0.05).

| **Dataset** | **Explanatory variable** | **Response variable** | **F value** | **p-value** |
| --- | --- | --- | --- | --- |
| UKBMS | Biotype specialism | Change in synchrony 1985-2000 | 14.97 | 0*** |
|  |  | Change in synchrony 2000-2012 | 39.11 | 0*** |
|  | Mobility | Average synchrony | 8.94 | 0.004** |
|  |  | Change in synchrony 2000-2012 | 453.98 | 0*** |
|  | Non-significant abundance | Change in synchrony 2000-2012 | 81.9 | 0*** |
|  | Significant abundance | Change in synchrony 1985-2000 | 5.06 | 0.016* |
|  |  | Change in synchrony 2000-2012 | 13.65 | 0*** |
| BBS | Biotype specialism | Change in synchrony | 4.05 | 0.049* |
|  | Mobility | Change in synchrony | 13.73 | 0*** |
| CBC | Biotype specialism | Change in synchrony | 11.88 | 0.002** |
|  | Abundance | Average synchrony | 7.42 | 0.004** |
|  | Significant abundance | Change in synchrony | 3.87 | 0.048* |
| **Significance is as follows: ***p < .001, **p < .01, *p < .05.** | | | | |

**Supplementary Material Appendix 2**

**Mixed effects models**

*All species model*

The ‘all species model’ (discussed in the section ‘Accounting for climatic synchrony’ in the main text) has population synchrony as the response variable, and year, mean northing, habitat similarity, distance and the selected climate synchrony variable(s) as fixed effects, and site pair ID and species as random effects. This was fitted for each dataset separately.

Eq. A1 $Y_{ij}= \beta_{0}+ \beta_{1}X_{1ij}+ \beta_{2}X_{2ij}+ \beta_{3}X_{3ij}+ \beta_{4}X_{4ij}+ \beta_{5}X_{5ij}+ \mu_{i}+v_{j}+ \varepsilon_{ij}$

where $i$ refers to the site pair ID and $j$ to species and $Y$ is the outcome variable, population synchrony. The model contains a parameter for intercept, $\beta_{0}$, parameters for the slope of the fixed effects, $\beta_{1}$ (year), $\beta_{2}$ (mean northing), $\beta_{3}$ (habitat similarity), $\beta_{4}$ (distance) and $\beta_{5}$ (climate synchrony variable(s)), random intercepts for both site pair ID and species, $\mu_{i}$ and $v_{j}$, and residual error term, $\varepsilon_{ij}$.

To understand whether mobility-attributes could explain differences in population synchrony, we fitted a variant of the ‘all species model’ for each dataset (discussed in the ‘Population synchrony and species attributes’ section in the main text). Firstly, we included each mobility attribute as a fixed effect.

Eq. A2 $Y_{ij}= \beta_{0}+ \beta_{1}X_{1ij}+ \beta_{2}X_{2ij}+ \beta_{3}X_{3ij}+ \beta_{4}X_{4ij}+ \beta_{5}X_{5ij}+ \beta_{6}X_{6ij}+ \mu_{i}+v_{j}+ \varepsilon_{ij}$

where $\beta_{6}$ is the mobility attribute of interest (specialism, mobility or mean abundance).

Secondly, we included an interaction between each mobility attribute and year to determine whether certain types of species increase or decrease in population synchrony over time.

Eq. A3 $Y_{ij}= \beta_{0}+ \beta_{1}X_{1ij}+ \beta_{2}X_{2ij}+ \beta_{3}X_{3ij}+ \beta_{4}X_{4ij}+ \beta_{5}X_{5ij}+ \beta_{6}X_{6ij}+ \beta_{7}X_{1ij}X_{6ij}+ \mu_{i}+v_{j}+ \varepsilon_{ij}$

where $\beta_{7}$ is the interaction between the mobility attribute of interest and year.
